# Nickel–cobalt oxalate as an efficient non-precious electrocatalyst for an improved alkaline oxygen evolution reaction†

**DOI:** 10.1039/d1na00034a

**Published:** 2021-04-27

**Authors:** Sourav Ghosh, Rajkumar Jana, Sagar Ganguli, Harish Reddy Inta, Gouri Tudu, Heramba V. S. R. M. Koppisetti, Ayan Datta, Venkataramanan Mahalingam

**Affiliations:** Department of Chemical Sciences, Indian Institute of Science Education and Research (IISER) Kolkata Mohanpur West Bengal 741246 India mvenkataramanan@yahoo.com sourav.g1989@gmail.com; Technical Research Centre, S. N. Bose National Centre for Basic Sciences Block-JD, Sector-III, Salt Lake Kolkata-700106 India; School of Chemical Sciences, Indian Association for the Cultivation of Science Jadavpur Kolkata-700032 India spad@iacs.res.in

## Abstract

The quest for developing next-generation non-precious electrocatalysts has risen in recent times. Herein, we have designed and developed a low cost electrocatalyst by a ligand-assisted synthetic strategy in an aqueous medium. An oxalate ligand-assisted non-oxide electrocatalyst was developed by a simple wet-chemical technique for alkaline water oxidation application. The synthetic parameters for the preparation of nickel–cobalt oxalate (Ni_2.5_Co_5_C_2_O_4_) were optimized, such as the metal precursor (Ni/Co) ratio, oxalic acid amount, reaction temperature, and time. Microstructural analysis revealed a mesoporous block-like architecture for nickel–cobalt oxalate (Ni_2.5_Co_5_C_2_O_4_). The required overpotential of Ni_2.5_Co_5_C_2_O_4_ for the alkaline oxygen evolution reaction (OER) was found to be 330 mV for achieving 10 mA cm_geo_^−2^, which is superior to that of NiC_2_O_4_, CoC_2_O_4_, NiCo_2_O_4_ and the state-of-the-art RuO_2_. The splendid performance of Ni_2.5_Co_5_C_2_O_4_ was further verified by its low charge transfer resistance, impressive stability performance, and 87% faradaic efficiency in alkaline medium (pH = 14). The improved electrochemical activity was further attributed to double layer capacitance (*C*_dl_), which indefinitely divulged the inferiority of NiCo_2_O_4_ compared to Ni_2.5_Co_5_C_2_O_4_ for the alkaline oxygen evolution reaction (OER). The obtained proton reaction order (*ρ*_RHE_) was about 0.80, thus indicating the proton decoupled electron transfer (PDET) mechanism for OER in alkaline medium. Post-catalytic investigation revealed the formation of a flake-like porous nanostructure, indicating distinct transformation in morphology during the alkaline OER process. Further, XPS analysis demonstrated complete oxidation of Ni^2+^ and Co^2+^ centres into Ni^3+^ and Co^3+^, respectively under high oxidation potential, thereby indicating active site formation throughout the microstructural network. Additionally, from BET-normalised LSV investigation, the intrinsic activity of Ni_2.5_Co_5_C_2_O_4_ was also found to be higher than that of NiCo_2_O_4_. Finally, Ni_2.5_Co_5_C_2_O_4_ delivered a TOF value of around 3.28 × 10^−3^ s^−1^, which is 5.56 fold that of NiCo_2_O_4_ for the alkaline OER process. This report highlights the unique benefit of Ni_2.5_Co_5_C_2_O_4_ over NiCo_2_O_4_ for the alkaline OER. The structure–catalytic property relationship was further elucidated using density functional theory (DFT) study. To the best of our knowledge, nickel–cobalt oxalate (Ni_2.5_Co_5_C_2_O_4_) was introduced for the first time as a non-precious non-oxide electrocatalyst for alkaline OER application.

## Abstract 2

Nickel–cobalt oxalate (Ni_2.5_Co_5_C_2_O_4_–*n*H_2_O) based block-like nanostructure has been introduced as superior electrocatalyst compared to nickel–cobalt oxide (NiCo_2_O_4_) for alkaline water oxidation.

## Graphical abstract

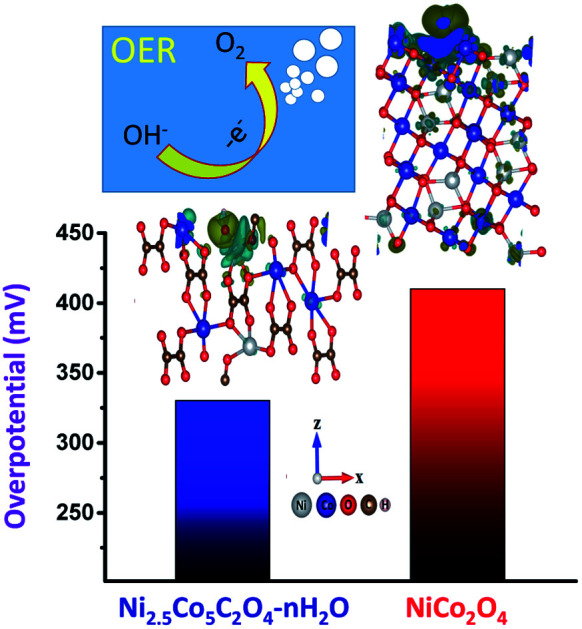

## 1. Introduction

Water splitting is becoming the most promising approach for hydrogen fuel production and energy storage.^1–5^ The requirement of a large overpotential for four electron trajectories all through the oxygen evolution reaction (OER) restricts the application in bulk mode.^6,7^ The ideal category of catalyst for OER is still RuO_2_ in alkaline and IrO_2_ in acidic solution.^8^ However, the cost of scalable design and availability in nature limit the use of such precious elements in day to day industrial applications. Non-precious transition metal based electrocatalysts particularly oxide and hydroxide have attracted great interest particularly owing to their simple synthetic strategy, extraordinary OER activity, and cheap market price.^9,10^ The improved alkaline OER performance can be attributed to the corrosion resistant physiochemical properties of the working electrode leading to substantial stability in alkaline medium.^11^ However, non-precious non-oxide transition metal based electrocatalysts are prone to quick *in situ* electrochemical active surface transformation, thereby rendering rapid kinetics, a low overpotential barrier and high stability. The performance of this catalytic system originates from the presence of microstructural defects, porosity and pore architecture, conductivity and oxidation (chemical) states of active elements.^12–15^ For example, non-oxide electrocatalysts based on cobalt (Co) are well-known for their metallic nature, which encourages their wide practical application.^16–18^ There are quite a few literature studies on non-precious non-oxide electrocatalysts for alkaline OER application, such as Co_4_N, (Co_0.7_Fe_0.3_)_2_B, (Co_*x*_Fe_1−*x*_)_2_P and Co_0.85_Se/Co_9_Se_8_.^19–22^ Thus, non-precious non-oxide electrocatalysts have attracted great attention for the alkaline OER.

A ligand assisted soft-chemical strategy has been employed for the design of electrocatalysts in recent years. Feng *et al.* demonstrated an ionic liquid assisted microwave irradiation technique for the preparation of nickel–cobalt fluoride for water electrolysis.^23^ Next, hierarchical hydrous cobalt phosphate micro-flower was employed as an efficient electrocatalyst for the alkaline OER.^24^ Recently, our group has discussed the superior OER electrocatalytic activity of NiCo_2_O_*x*_S_4−*x*_ over NiCo_2_O_4_.^25^ On the other hand, transition metal based oxalate is renowned as a carbon sink, suggesting it as a more sustainable way to design energy storage materials. Numerous bio- and artificial-synthesis routes have already been reported for the synthesis of oxalate anion (C_2_O_4_^2−^) from CO_2_.^26^ Therefore, utilization of oxalate-based materials is an impactful way from the clean and green synthesis viewpoint. To our knowledge, there are only few reports on oxalate based materials for OER applications. For instance, cobalt oxalate has shown promising activity as an electrocatalyst for alkaline OER applications.^27,28^ Liu *et al.* reported the most convenient and widely used solvothermal synthetic procedure for the preparation of CoC_2_O_4_–2H_2_O with a tuneable rod and 3-D polyhedron like morphology. Post-catalytic transformation into CoOOH species resulted in active sites for the alkaline OER with 436 and 492 mV overpotential (for 10 mA cm_geo_^−2^) for the micro-rod and 3-D polyhedron respectively.^29^ However, the high overpotential and sluggish kinetics of the catalyst are identified as major limitations for practical implementation in large scale production. To date, no such reports were found for nickel–cobalt oxalate, which might be an excellent electrocatalyst for alkaline water oxidation. In addition, fabrication of non-precious non-oxide transition metal based electrocatalysts that meet the requirement of 10 mA cm_geo_^−2^ current density, thereby reaching a benchmark of 12% solar-to-hydrogen efficiency, is a challenging task.

Herein, we have demonstrated an oxalic acid assisted non-precious non-oxide transition metal (nickel and cobalt) based electrocatalyst for the alkaline OER. Nickel–cobalt oxalate was primarily synthesized by a wet chemical technique, followed by annealing to form nickel–cobalt oxide. The preparation technique was optimised in terms of Ni/Co ratio, oxalic acid amount, temperature and processing time. The overpotential requirement for the block-like Ni_2.5_Co_5_C_2_O_4_ was found to be 330 mV for reaching the benchmark of 10 mA cm_geo_^−2^. The improved performance of the catalyst was further verified by low charge transfer resistance, long term stability and 87% faradic efficiency. Interestingly, the control studies and the change in double layer capacitance (*C*_dl_) because of electrochemical preconditions clearly suggest that the alkaline OER catalytic performance of Ni_2.5_Co_5_C_2_O_4_ is superior to that of its high temperature oxide counterpart *i.e.* NiCo_2_O_4_. The structure–catalytic property relationship was further elucidated by density functional theory (DFT) study which was performed to get insight into the improved electrochemical performance of Ni_2.5_Co_5_C_2_O_4_ compared to the CoC_2_O_4_, NiC_2_O_4_ and NiCo_2_O_4_, and atomic interpretation was also proposed from computed results. In addition, electrocatalytic measurements in electrolyte solutions with different pH values were performed to understand the mechanistic details of Ni_2.5_Co_5_C_2_O_4_ for the alkaline OER. This investigation exclusively unlocks a strategy to fabricate oxalate ligand assisted non-precious nickel–cobalt based bimetallic electrocatalysts for water oxidation application in alkaline medium.

## 2. Results and discussion

### 2.1 Structural characterization

**Fig. 1:**
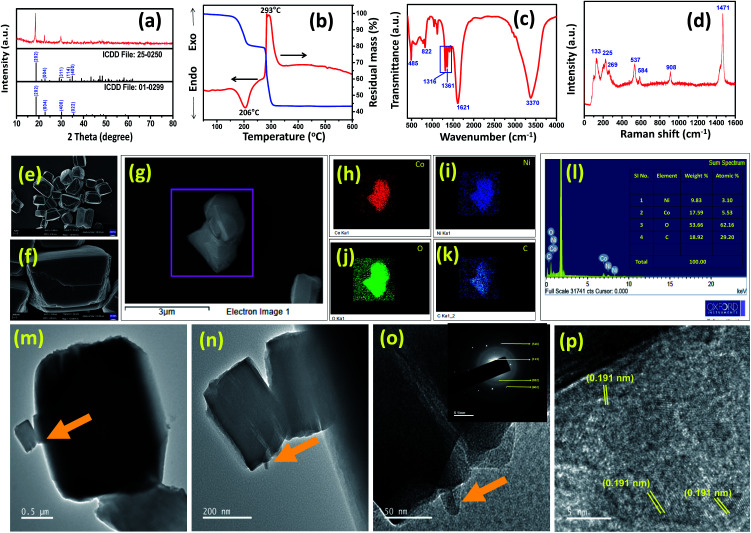
(a) PXRD with the standard patterns of NiC_2_O_4_–2H_2_O (ICDD no. 01-0299) and CoC_2_O_4_–2H_2_O (ICDD no. 25-0250), (b) DTA-TG, (c) FTIR and (d) Raman pattern of the as-prepared nickel–cobalt oxalate (Ni_2.5_Co_5_C_2_O_4_) sample. Morphological study including (e and f) FESEM images, (g–k) elemental mapping, (l) EDS study, (m–o) TEM images (the inset showing SAED) and (p) HRTEM image of the as-prepared nickel–cobalt oxalate (Ni_2.5_Co_5_C_2_O_4_) sample.

The synthesis of nickel oxalate (NiC_2_O_4_), cobalt oxalate (CoC_2_O_4_) and nickel–cobalt oxalate (Ni_2.5_Co_5_C_2_O_4_) samples was performed by a one-pot method using metal nitrate salts and oxalic acid in aqueous media. For comparative study, nickel cobalt oxide (NiCo_2_O_4_) was prepared by calcination of nickel–cobalt oxalate (Ni_2.5_Co_5_C_2_O_4_) at 350 °C for 2 h (see the details in the Experimental and Characterization section, ESI, Table S1†). The elemental phase was examined by the X-ray diffraction (XRD) method. As shown in Fig. 1a, the peak position resembles with the appearance of cobalt oxalate hydrate (CoC_2_O_4_–2H_2_O; ICDD no. #25-0250) and nickel oxalate hydrate (NiC_2_O_4_–2H_2_O; ICDD no. #01-0299). The XRD pattern of Ni_2.5_Co_5_C_2_O_4_ is quite identical to that of phase-pure cobalt oxalate hydrate, thereby indicating the existence of substituted nickel at some portion of cobalt sites throughout the mixed-metal oxalate crystal framework.^30^ Furthermore, the XRD pattern of a physical mixture comprising CoC_2_O_4_ and NiC_2_O_4_ was examined, revealing doublet peaks at 35.1° and 35.59° that can be labelled as CoC_2_O_4_ and NiC_2_O_4_ respectively. However, Ni_2.5_Co_5_C_2_O_4_ shows a single broad peak around 34.9°, manifesting the (022) plane of CoC_2_O_4_. The resemblance of XRD patterns between Ni_2.5_Co_5_C_2_O_4_ and CoC_2_O_4_ further supports the existence of substituted nickel at some portion of cobalt sites throughout the mixed-metal oxalate crystal framework. In addition, the (022) peak of Ni_2.5_Co_5_C_2_O_4_ shifts to a lower 2*θ* value w.r.t CoC_2_O_4_, revealing [022] oriented unit cell expansion due to the presence of the nickel component within the composite network (see Fig. S1, ESI†).^31^

Thermal stability of the as-prepared sample was corroborated by DTA-TG analysis. Fig. 1b shows an endothermic peak around 205 °C, indicating the removal of lattice water, resulting in 19% weight loss. The exothermic peak around 294 °C represents the decomposition of oxalate ligand with a 57% weight loss compared to the as-prepared mixed oxalate sample.^32^ No further weight loss is observed in the 300 to 600 °C temperature range. Thus, for the preparation of the NiCo_2_O_4_ sample, the as-prepared Ni_2.5_Co_5_C_2_O_4_ was calcined at 350 °C. Fourier-transform infrared (FTIR) spectroscopy study was carried out for further structural characterization of the sample (Fig. 1c). The FTIR peak positioned around 485 cm^−1^ is assigned to metal–oxygen vibration mode. The C–O vibration mode is obtained around 1316 (symmetric) and 1361 cm^−1^ (asymmetric) along with a strong vibrational band at 1621 cm^−1^, which is a signature characteristic peak for carbonyl stretching vibrational mode, confirming the presence of the oxalate moiety in the as-prepared sample.^29^ The broad vibrational band around 3370 cm^−1^ is attributed to water molecules, while 822 cm^−1^ is to asymmetric O–C–O vibrational mode. Raman measurements were also performed to obtain more structural information (Fig. 1d). The intense peak around 912 cm^−1^ is attributed to C–C stretching mode, which is IR-silent. The presence of C–C–O bending mode is concluded from the observed 532 cm^−1^ peak. In addition, appearance of peaks around 225 and 266 cm^−1^ is ascribed to M–O stretching and O–M–O ring-bending modes respectively, where M stands for the metal centre (cobalt and nickel).^16,33^ The XPS technique was employed for the analysis of chemical state and probable elemental composition of the as-prepared sample (see Fig. S2, ESI†). The C 1s spectrum exhibits peaks around 286.48, 287.69 and 290.3 eV particularly for C–O, C=O and C–F respectively (see Fig. S2a, ESI†). The appearance of 290.3 eV binding energy could originate from Nafion residue which is used as a binder.^34,35^ As shown in Fig. S2b (ESI†), peaks at 782.93 and 798.87 eV can be designated as Co 2p_3/2_ and Co 2p_1/2_, respectively with a clear separation of 15.94 eV, confirming the presence of Co^2+^.^36^ The satellite signals around 786.79 and 803.86 eV further support the same (see Fig. S2b, ESI†). The presence of Ni^2+^ can be suspected from the binding energy peaks around 857.71 and 875.35 eV assigned to Ni 2p_3/2_ and Ni 2p_1/2_ respectively, along with satellite peaks at 862.61 and 881.14 eV (see Fig. S2c, ESI†).^37^ The narrow scan of the O 1s peak is deconvoluted into two peaks at 533.62 and 534.65 eV representing C–O and lattice water molecules, respectively (see Fig. S2d, ESI†).^38^ Inductively coupled plasma-atomic emission spectroscopy (ICP-AES) was also employed to get quantitative information regarding the elemental composition of Ni_2.5_Co_5_C_2_O_4_. The Ni/Co ratio is found to be 1 : 2.018 (see Table S2, ESI†).

### 2.2 Morphological characterization

Microstructural analysis was performed by FESEM and TEM investigation. The displayed FESEM image presents block shaped nanostructures of Ni_2.5_Co_5_C_2_O_4_ (Fig. 1e and f). Close inspection reveals a smooth surface along with a clear boundary, thereby indicating sturdy close-packed interaction among the crystallites. Elemental distribution was further determined by energy-dispersive X-ray spectroscopy (EDS) and mapping investigation (Fig. 1g–l). It shows uniform spreading of Ni and Co throughout the framework with a Ni/Co ratio of about 1 : 2. TEM study also shows a block-like morphology, which is formed from self-assembly of porous sheet-like nanostructures (Fig. 1m–o). The selected area electron diffraction (SAED) image indeed shows crystalline features of the sample which is further confirmed by 0.191 nm interplanar distance, indicating the (602) plane from HRTEM analysis (Fig. 1o and p). To study the morphological evolution, time dependent synthesis was carried out at different time intervals from 15 min to 60 min (see Fig. S3, ESI†). However, no specific change in morphology was observed from 15 min to 2 h. As a control experiment, phase pure CoC_2_O_4_, NiC_2_O_4_ and NiCo_2_O_4_ samples were prepared and subjected to microstructural analysis. Fig. S4a–c (ESI†) shows the nano-rod like architecture of CoC_2_O_4_ with a smooth surface. The TEM image presents a controllable 1-D microstructure with 150–250 nm diameter (see Fig. S5a–c, ESI†). The HRTEM image indicates 0.296 nm lattice fringes for the (400) plane (see Fig. S3d, ESI†). NiC_2_O_4_ reveals an irregular sheet-like architecture with a packed-surface, thus further indicating that nano-block units self-assembled to grow such an architecture (see Fig. S6a–c, ESI†). TEM investigation demonstrates the formation of sheet-like nanostructures with 0.390 nm interplanar spacing of the (004) plane (see Fig. S7a–d, ESI†). For comparison, NiCo_2_O_4_ was synthesized by high-temperature thermal treatment of the as-prepared Ni_2.5_Co_5_C_2_O_4_. The XRD pattern confirms the formation of phase pure NiCo_2_O_4_ and absence of any impurity phase such as NiO and Co_3_O_4_ (see Fig. S8a, ESI†). FESEM study reveals a block-like nano-porous network, thus indicating the formation of porosity due to thermal decomposition of oxalate during the high temperature treatment (see Fig. S8b and c, ESI†). EDS and elemental mapping indicate the distribution of Ni and Co throughout the framework with a Ni/Co ratio of about 1 : 2, which is further supported by ICP-AES analysis (see Fig. S8d–j, Table S3, ESI†). The TEM image indicates a micro-sheet like framework with 0.234 and 0.245 nm lattice fringes for (222) and (311) planes respectively (see Fig. S9a–d, ESI†). Close inspection shows that small nanoparticles assembled together to form a micro-sheet like architecture, leading to uniform distribution of interparticle porosity.

### 2.3 Electrochemical study

**Fig. 2:**
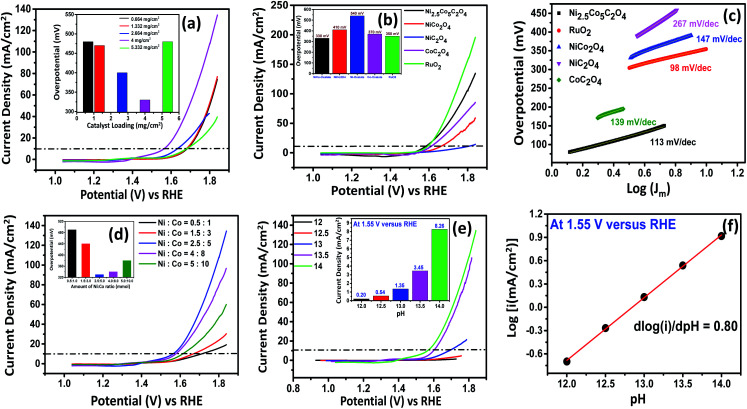
(a) *iR*-corrected LSV scan for different loading amounts for Ni_2.5_Co_5_C_2_O_4_, (b) *iR*-corrected LSV scan for different electrocatalysts [the inset showing the overpotential value to reach the benchmark of 10 mA cm_geo_^−2^], (c) Tafel slope analysis of different electrocatalysts, (d) *iR*-corrected LSV scan for different Ni/Co ratios, (e) LSV scan and (f) linear fit of Ni_2.5_Co_5_C_2_O_4_ with the variation of pH.

To investigate the electrochemical activity, working electrodes were prepared with different loading amounts of Ni_2.5_Co_5_C_2_O_4_ by the drop-casting technique. As shown in Fig. 2a, the best performance is obtained for 4 mg cm_geo_^−2^ catalyst loading using carbon paper as the substrate. For the benchmark value of 10 mA cm_geo_^−2^, Ni_2.5_Co_5_C_2_O_4_ consumes 330 mV overpotential for 4 mg cm^−2^ catalyst loading (see Table S4, ESI†).^39^ However, the catalytic activity of the carbon paper supported electrocatalyst was also compared to that obtained with glassy carbon as the substrate. As displayed in Fig. S10,† the glassy carbon drop-cast electrocatalyst showed lower mass activity at 1.7 V *vs.* RHE, showing the contribution from the porous network of carbon paper that could lead to better electrolyte diffusion for improved electrocatalytic performance of the Ni_2.5_Co_5_C_2_O_4_ sample. However, a comparative study was also carried out with this optimized loading of 4 mg cm_geo_^−2^ for carbon paper supported reference samples such as CoC_2_O_4_, NiC_2_O_4_ and standard RuO_2_. To investigate the electrochemical features, the CV technique was used with correction for uncompensated solution resistance (*R*_u_) for all the electrocatalysts (Fig. S11†). The overpotential to reach the benchmark of 10 mA cm_geo_^−2^ is 370, 540 and 350 mV for CoC_2_O_4_, NiC_2_O_4_ and standard RuO_2_, respectively (Fig. 2b). Again, the OER activity of Ni_2.5_Co_5_C_2_O_4_ is superior compared to that of NiCo_2_O_4_, which requires 410 mV overpotential for reaching 10 mA cm_geo_^−2^. The Tafel plot was recorded to study the OER kinetics of electrocatalysts. Fig. 2c shows a lower value of the Tafel slope for Ni_2.5_Co_5_C_2_O_4_ (81 mV dec^−1^) compared to control samples, such as CoC_2_O_4_ (90 mV dec^−1^), NiC_2_O_4_ (229 mV dec^−1^) and NiCo_2_O_4_ (121 mV dec^−1^), suggesting the relatively rapid kinetics for Ni_2.5_Co_5_C_2_O_4_ compared to others.^35^ However, the state-of-the-art RuO_2_ electrocatalyst exhibited 79 mV dec^−1^ Tafel slope, which is the best among all the samples (see Table S5, ESI†). Composition optimization was performed with the variation of Ni/Co amount, and the best activity was observed for a Ni/Co ratio of about 2.5/5, which is indexed as Ni_2.5_Co_5_C_2_O_4_ (see Fig. 2d, S12a and Table S6, ESI†). Thereafter, the process parameter for the synthesis of the Ni_2.5_Co_5_C_2_O_4_ catalyst was further optimized, such as the amount of oxalic acid, reaction temperature and time. The best performance was obtained with 12 mmol oxalic acid, 80 °C reaction temperature and 2 hours reaction time (see Fig. S12b–d and Tables S7–S9, ESI†). In addition, the OER mechanism for Ni_2.5_Co_5_C_2_O_4_ was investigated with the variation of pH of electrolytes (12, 12.5, 13, 13.5 and 14) under alkaline conditions (Fig. 2e). Proton reaction order is considered as the most convenient parameter to study the mechanistic pathway and estimated from the following equation:
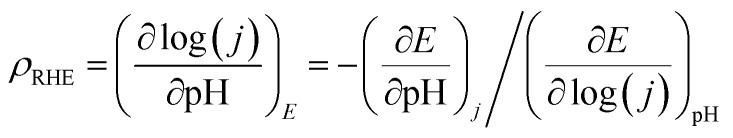
where *ρ*_RHE_ refers to the proton reaction order on the RHE scale.^40^ The result suggests that the proton reaction order (*ρ*_RHE_) for the Ni_2.5_Co_5_C_2_O_4_ is 0.80, thus proposing proton decoupled electron transfer (PDET) trajectory for the alkaline OER process (Fig. 2f). The deprotonation pathway is presumed to proceed as follows: OOH_(ads)_ + OH^−^ → OO_(ads)_ + H^+^ + e^−^ and the decoupling assisted deprotonation mechanism is most consistent with it.^41^

**Fig. 3:**
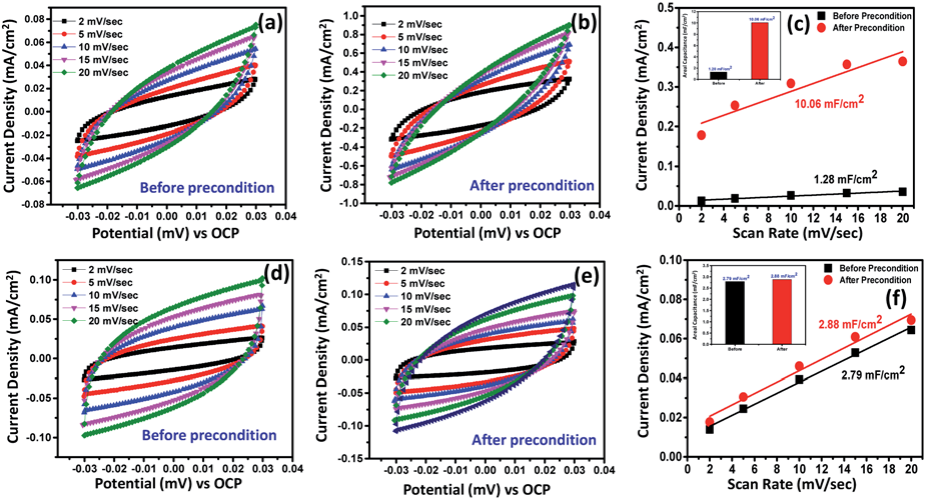
CV curves recorded at different scan rates to estimate the double layer capacitance (*C*_dl_) for (a and b) Ni_2.5_Co_5_C_2_O_4_ and (d and e) NiCo_2_O_4_. Linear fit for the estimation of double layer capacitance (*C*_dl_) from the DLC current *vs.* scan rate plot for (c) Ni_2.5_Co_5_C_2_O_4_ and (f) NiCo_2_O_4_.

Fig. S13† presents the magnified CV data for both Ni_2.5_Co_5_C_2_O_4_ and NiCo_2_O_4_ samples. From the as-displayed oxidation and reduction peaks, higher current density and peak area are noted for the Ni_2.5_Co_5_C_2_O_4_ compared to NiCo_2_O_4_. This also indicates that improved geometric OER performance may be attributed to the presence of more number of accessible active sites in the alkaline medium for the Ni_2.5_Co_5_C_2_O_4_ sample.^42^ Further, the requirement of 10 mA cm^−2^ was achieved at 1.56 V *vs.* RHE for Ni_2.5_Co_5_C_2_O_4_, whereas NiCo_2_O_4_ delivered 1.64 V *vs.* RHE for the same. It unequivocally implies the higher geometrical OER activity of Ni_2.5_Co_5_C_2_O_4_ than NiCo_2_O_4_. To gather further support, double layer capacitance (*C*_dl_) was calculated that was indefinitely assumed as a non-destructive parameter for the electrochemical active surface area (ECSA) calculations.^43^ The *C*_dl_ value for Ni_2.5_Co_5_C_2_O_4_ is calculated to be 1.31 mF cm^−2^, whereas NiCo_2_O_4_ shows 2.84 mF cm^−2^ just before precondition (Fig. 3a and d). However, the *C*_dl_ value is significantly increased up to 10.61 mF cm^−2^ after electrochemical precondition for Ni_2.5_Co_5_C_2_O_4_, while the corresponding value for NiCo_2_O_4_ remains nearly the same at 2.93 mF cm^−2^ (Fig. 3b and e). Thus development of a higher number of accessible active sites is observed during electrochemical precondition for Ni_2.5_Co_5_C_2_O_4_ compared to the NiCo_2_O_4_ sample (Fig. 3c and f).^44^ This also suggests more ECSA and thus more surface coverage (*θ*) that provides a higher value of current (*j*) according to the following expression:
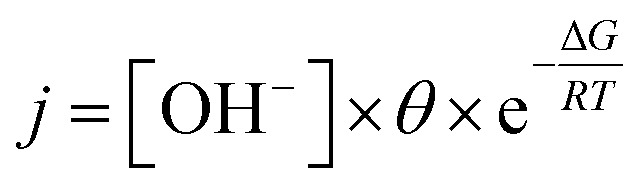
where the current (*j*) value is directly proportional to surface coverage (*θ*) involving *OOH or *OH sites, [OH^−^] concentration and exponential factor which actually depends on the adsorbed surface of intermediate species for the alkaline OER.^40^ The change in *C*_dl_ is also estimated for CoC_2_O_4_ and NiC_2_O_4_ samples that further supports the better electrochemical performance of CoC_2_O_4_ compared to NiC_2_O_4_ for the alkaline OER (see Fig. S14 and S15, ESI†).

**Fig. 4:**
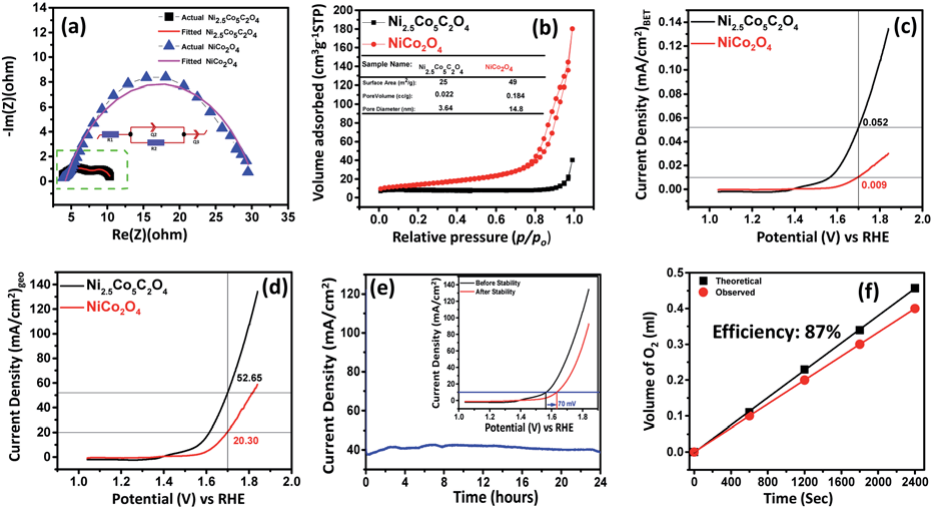
(a) Nyquist plots of different electrocatalysts, (b) N_2_ adsorption–desorption isotherm of Ni_2.5_Co_5_C_2_O_4_ and NiCo_2_O_4_ samples, catalytic performance in terms of (c) BET surface area normalised LSV plot and (d) geometrical area normalised LSV plot for Ni_2.5_Co_5_C_2_O_4_ and NiCo_2_O_4_ samples, (e) stability study of the best catalyst Ni_2.5_Co_5_C_2_O_4_ [the inset showing the overpotential value to reach the benchmark of 10 mA cm_geo_^−2^], and (f) analysis of faradaic efficiency for the Ni_2.5_Co_5_C_2_O_4_.

To further study the electrochemical properties, electrochemical impedance spectroscopy (EIS) was employed to evaluate the charge transfer resistance (*R*_ct_) and transport kinetics. As expected, Ni_2.5_Co_5_C_2_O_4_ delivers a lower R_ct_ value of about 6.34 ohm in comparison with NiCo_2_O_4_ (26.14 ohm), thereby indicating the occurrence of rapid charge transport phenomena for Ni_2.5_Co_5_C_2_O_4_ (Fig. 4a).^45^ Likewise, NiC_2_O_4_ shows a higher *R*_ct_ value than CoC_2_O_4_, justifying the higher conducting behaviour of CoC_2_O_4_ in alkaline medium (see Fig. S16, ESI†). To study the intrinsic activity of the drop-cast catalyst, BET normalized electrochemical performance was already reported as the most convenient analytical technique by Shao-Horn and co-workers.^46^ As shown in Fig. 4b, the nitrogen adsorption–desorption isotherm displays a type-IV isotherm and H-3 hysteresis, reflecting a mesoporous network and slit-like pore geometry respectively.^47^ The BET surface area is found to be 25 and 49 m^2^ g^−1^ for Ni_2.5_Co_5_C_2_O_4_ and NiCo_2_O_4_ respectively, which could be attributed to thermal decomposition of oxalate ligand during calcination treatment at high-temperature. Fig. 4c presents the comparative survey of intrinsic alkaline OER performance for both the samples. From the BET normalized LSV plot, Ni_2.5_Co_5_C_2_O_4_ shows a current density of about 0.052 mA cm_BET_^−2^, which is 5.7 fold greater than that of NiCo_2_O_4_ at 1.7 V *vs.* RHE. In addition, the geometrical performance of Ni_2.5_Co_5_C_2_O_4_ is also superior to that of NiCo_2_O_4_ for the OER in alkaline medium (Fig. 4d). This result indicates the superiority of Ni_2.5_Co_5_C_2_O_4_ over its widely used high temperature calcined product of NiCo_2_O_4_ from both intrinsic and geometrical aspects for the OER under alkaline conditions.^48^ To evaluate the applicability for long-term usage, the stability test of the Ni_2.5_Co_5_C_2_O_4_ catalyst was performed for 24 hours by the chronoamperometry technique (Fig. 4e). Alkaline OER performance is relatively stable up to the initial 12 hours of the experiment; thereafter, the performance slightly decreased with time. After the stability test, the Ni_2.5_Co_5_C_2_O_4_ sample achieves 10 mA cm_geo_^−2^ with around 350 mV overpotential, which is roughly 20 mV more than that of the as-prepared electrocatalyst (inset of Fig. 4e). Interestingly, the Ni/Co ratio is found to be 1 : 2.38, indicating mechanical inertness of the electrocatalyst even after the long-term 24 h stability test at pH = 14.

Finally, faradaic efficiency was studied and the obtained value for Ni_2.5_Co_5_C_2_O_4_ is 87% (Fig. 4f). It indeed denotes sufficiently high activity of the electrocatalyst for alkaline water oxidation reaction. Turnover frequency (TOF) is one of the essential parameters to examine the intrinsic performance of the electrocatalyst. The TOF is expressed as follows:
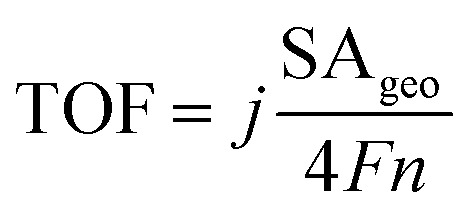
where *F*, *n* and *j* denote the Faraday constant (96 485C mol^−1^), moles of the catalytically active species and geometrical area (SA_geo_) normalized current density (mA cm^−2^)_geo_.^49^ The amount of active elemental species was calculated from ICP-AES study (see Tables S2 and S3, ESI†). The TOF value is found to be 3.28 × 10^−3^ s^−1^ for Ni_2.5_Co_5_C_2_O_4_, whereas the value for NiCo_2_O_4_ is 5.89 × 10^−4^ s^−1^ at 1.63 V *vs.* RHE, indicating 5.56 fold increased intrinsic superiority of Ni_2.5_Co_5_C_2_O_4_ over NiCo_2_O_4_, which is also previously supported by BET normalised LSV investigation. Furthermore, the electrochemical activity of Ni_2.5_Co_5_C_2_O_4_ is almost found to be comparable with that of earlier reported non-precious electrocatalysts and standard RuO_2_ for the alkaline OER (see Table S10, ESI†).^19–24,29,50–53^

### 2.4 Post-catalytic analysis

To understand the post-catalytic transformation, the carbon paper supported catalyst was subjected to composition and microstructural analysis. As evident from Fig. S17 (ESI†), no characteristic XRD pattern of the post-catalytic sample is observed except the sharp conspicuous diffraction signal which is assigned to the peaks from carbon paper. However, XPS measurements clearly show the structural features and elemental composition of the post-catalytic sample (see Fig. S18, ESI†). The C 1s pattern shows the appearance of two new signals around 291.77 and 294.76 eV, indicating the existence of a K^+^ adsorbed microstructural framework for the post-catalytic sample (see Fig. S18a, ESI†).^37^ From the Co 2p narrow scan, the peaks around 780.25 and 796.15 eV could corroborate the existence of Co^3+^ in the post-catalytic network (see Fig. S18b, ESI†).^36^ Further, the presence of satellite peaks around 785.33 and 802.35 eV confirms the same.^54^ Likewise, distinct XPS signals around 855.95 and 874.1 eV along with satellite peaks around 861.54 and 880.01 eV confirm the presence of Ni^3+^ (see Fig. S18c, ESI†).^37^ However, the O 1s spectrum shows peaks around 530.01, 531.08 and 534.69 eV particularly for lattice oxygen, hydroxyl groups (adsorbed) and lattice water molecules respectively (see Fig. S18d, ESI†). Thus, composition analysis clearly shows that almost all Ni^2+^ and Co^2+^ oxidizes into Ni^3+^ and Co^3+^ respectively under high oxidation potential gradient during the OER process. Furthermore, inductively coupled plasma-atomic emission spectroscopy (ICP-AES) study reveals a Ni/Co ratio of about 1 : 2.15 for the post-catalyst sample, thereby confirming the absence of any such metal ion leaching during the OER for Ni_2.5_Co_5_C_2_O_4_ that further confirms the mechanical stability of the working electrode in alkaline medium. The morphology of the post-OER sample was investigated by FESEM and TEM. The FESEM image shows flake-like assembly, which confirms structural transformation from a block to flake-like nanostructure under alkaline OER conditions (see Fig. S19a–d, ESI†). In addition, TEM investigation displays microstructural aggregation throughout the mesoporous network of the post-catalytic sample with lattice fringes of 0.220 nm representing the (006) plane of CoOOH (ICDD no. 014-0673).^48^ EDS and elemental mapping further reveal the uniform spreading of Ni, Co, C and O for the post-catalytic sample (see Fig. S19e–j, ESI†). For further validation, Raman measurements were performed to examine the *in situ* transformation of the catalyst during electrochemical precondition treatment. The Raman signals appear at 186, 470, 510 and 666 cm^−1^ for A_g_, E_g_, F_2g_ and A_1g_ respectively, confirming the formation of oxyhydroxide species from nickel cobalt oxalate (see Fig. S20, ESI†).^20,29^

### 2.5 Computational study

**Fig. 5:**
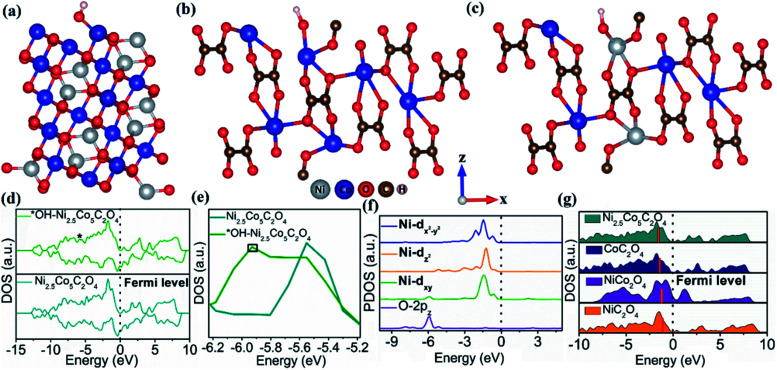
Optimized structure of OH* adsorbed (a) NiCo_2_O_4_ (311), (b) CoC_2_O_4_ (202), and (c) Ni_2.5_Co_5_C_2_O_4_ (202) surfaces, (d) DOS plots for Ni_2.5_Co_5_C_2_O_4_ (202) and OH*–Ni_2.5_Co_5_C_2_O_4_ (202) surfaces (asterisk indicates the contribution from O-2p), (e) magnified DOS (the O-2p state is situated at around −5.95 eV as marked by a black box in the OH*–Ni_2.5_Co_5_C_2_O_4_ (202) DOS), (f) projected density of states (PDOS) for O-2p_*z*_, Ni-d_*xy*_, Ni-d_*z*_^2^ and Ni-d_*x*_^2^_–*y*_^2^ of OH*–Ni_2.5_Co_5_C_2_O_4_ (202), and (g) DOS plots for different catalyst surfaces. The thick red bar indicates the position of the d-band centre (*E*_d_) on catalyst surfaces.

To get better insight into the structure–catalytic property relationship of the designed materials, we have performed density functional theory (DFT) study based on first-principles calculations using the Vienna *Ab Initio* Simulation Package (VASP).^55^ As revealed from PXRD analysis, the highest intensity planes namely (311) for NiCo_2_O_4_ and (202) for NiC_2_O_4_, CoC_2_O_4_ and Ni_2.5_Co_5_C_2_O_4_ are considered to be the active planes for the electrochemical OER and all the DFT studies were performed on NiCo_2_O_4_ (311), NiC_2_O_4_ (202), CoC_2_O_4_ (202) and Ni_2.5_Co_5_C_2_O_4_ (202) surfaces. The NiCo_2_O_4_ (311) surface contains surface exposed Ni^2+^ and Co^3+^ ions which are bi and tri-coordinated respectively while both NiC_2_O_4_ (202) and CoC_2_O_4_ (202) surfaces consist of tri-coordinated surface exposed Ni^2+^ and Co^2+^ respectively (see Fig. S21a–c, ESI†). On the contrary, the Ni_2.5_Co_5_C_2_O_4_ (202) surface contains both surface exposed Ni^2+^ and Co^2+^ (see Fig. S21d, ESI†). However, all of the oxalates have bulk tetra and penta-coordinated Ni^2+^ and Co^2+^ ions (see Fig. S21, ESI†). The M–OH bond strength for the metal-based electrocatalysts is considered to be the key descriptor for OER activity.^56–60^ The weaker the M–OH bond strength, the higher will be the activity. The more stable OH adsorption site for the NiCo_2_O_4_ (311) surface is Co^3+^ with a binding energy (B. E.) of −0.65 eV while those for NiC_2_O_4_ and CoC_2_O_4_ (202) surfaces are −1.15 and −0.46 eV respectively (see Fig. 5a–c, S22 and Table S11, ESI†). On the other hand, tri-coordinated surface Ni^2+^ ion is the most stable site for OH adsorption with a B.E. of −0.23 eV (see Fig. S23 and Table S11, ESI†). Hence, the M–OH bond strength follows the order Ni_2.5_Co_5_C_2_O_4_ (202) < CoC_2_O_4_ (202) < NiCo_2_O_4_ (311) < NiC_2_O_4_ (202). In addition, Bader charge analysis^61,62^ exhibits a charge transfer of 0.28|*e*| from adsorbed OH (OH*) to the Ni_2.5_Co_5_C_2_O_4_ (202) catalyst surface, indicating relatively weaker charge transfer interaction compared to CoC_2_O_4_ (0.4|*e*|), NiCo_2_O_4_ (0.65|*e*|) and NiC_2_O_4_ (0.84|*e*|) surfaces. The charge density difference (CDD) plot further confirms the charge transfer from OH* to different surfaces (Fig. S24†). Therefore, with relatively weaker M–OH bonding interaction, the Ni_2.5_Co_5_C_2_O_4_ (202) catalyst surface is superior to the other surfaces and consequently the order of OER activity is found to be NiC_2_O_4_ (202) < NiCo_2_O_4_ (311) < CoC_2_O_4_ (202) < Ni_2.5_Co_5_C_2_O_4_ (202), which very nicely corroborates the experimental findings (*vide infra*). The bonding interaction between adsorbed OH and interacting Ni in OH*–Ni_2.5_Co_5_C_2_O_4_ (202) can be further confirmed from the density of states (DOS) plot (asterisk indicates the contribution from O-2p) as shown in Fig. 5d. The generation of the O-2p state can be clearly visualized near −5.95 eV (as marked with a black box) in the DOS plot for OH*–Ni_2.5_Co_5_C_2_O_4_ (202) (Fig. 5e). Besides, projected density of states (PDOS) analysis further confirms the bonding interaction between O-2p_*z*_ and Ni-d_*xy*_ orbitals which indicates that hybridization mainly occurs between these orbitals (Fig. 5f).

From the above discussion, it can be concluded that the adsorption of OH is weaker on the Ni_2.5_Co_5_C_2_O_4_ (202) catalyst surface and hence exhibiting better OER activity and this conclusion can be further justified by a d-band model which correlates the d-band centre (DBC) with the adsorption energies of the intermediates (here OH).^63–65^ The positions of DBCs are −1.50, −1.37, −1.26 and −1.07 eV respectively for Ni_2.5_Co_5_C_2_O_4_ (202), CoC_2_O_4_ (202), NiCo_2_O_4_ (311) and NiC_2_O_4_ (202) surfaces (Fig. 5g) which vividly shows that the DBC gradually shifts towards the Fermi level from Ni_2.5_Co_5_C_2_O_4_ to the NiC_2_O_4_ (202) surface. The closer the DBC to the Fermi level, the stronger will be the adsorption of OH and hence deterioration of OER activity. This finding overwhelmingly supports our conclusion that the Ni_2.5_Co_5_C_2_O_4_ catalyst is the best surface for the OER with weaker OH adsorption ability while NiC_2_O_4_ is the least active with stronger OH binding ability. The present study highlights the benefit of nickel–cobalt oxalate over nickel–cobalt oxide for the alkaline OER process. We also believe that the use of oxalate ligand could be a promising strategy for the fabrication of new-generation electrocatalysts for electrochemical water oxidation in alkaline medium.

## 3. Conclusions

In summary, nickel–cobalt oxalate (Ni_2.5_Co_5_C_2_O_4_) based block-like nanostructures were prepared by a wet-chemical technique in an aqueous medium and further treated by calcination at 350 °C for the synthesis of nickel cobalt oxide (NiCo_2_O_4_). The preparation method was optimized in terms of Ni/Co ratio, oxalic acid amount, reaction temperature and time. The working electrode fabrication was performed on carbon paper as the substrate surface maintaining an optimum loading of the electrocatalyst. Next, Ni_2.5_Co_5_C_2_O_4_ achieves 10 mA cm_geo_^−2^ with 330 mV overpotential, whereas NiCo_2_O_4_ requires 410 mV for the same. The comparative study suggests the best electrochemical catalytic efficiency for Ni_2.5_Co_5_C_2_O_4_, and that can be attributed to lower charge transfer resistance, more changes in double layer capacitance during precondition in alkaline medium, and high surface coverage. DFT study reveals that comparatively weaker OH adsorption as well as favourable d-band centre (DBC) position makes the Ni_2.5_Co_5_C_2_O_4_ catalyst the best surface for the OER compared to individual CoC_2_O_4_ and NiC_2_O_4_ as well as well studied NiCo_2_O_4_. This report thoroughly compares the activity of Ni_2.5_Co_5_C_2_O_4_ and NiCo_2_O_4_ for the alkaline OER process and highlights the benefit of Ni_2.5_Co_5_C_2_O_4_ over NiCo_2_O_4_. The prudent choice of the oxalate based ligand-assisted synthetic approach could be used for other transition metals to execute bulk-scale alkaline water oxidation for a clean ecosystem and sustainable living.

## Conflicts of interest

There are no conflicts to declare.

## Supplementary Material

NA-003-D1NA00034A-s001
