# Supplementary material for: Nickel–cobalt oxalate as an efficient non-precious electrocatalyst for an improved alkaline oxygen evolution reaction
Source: Nanoscale Adv. 2021 Apr 27;3(13):3770–9. doi: 10.1039/d1na00034a (PMC9416859; doi:10.1039/d1na00034a)
Supplement: NA-003-D1NA00034A-s001 [file NA-003-D1NA00034A-s001.pdf]

Electronic Supplementary Material (ESI) for Nanoscale Advances.
This journal is © The Royal Society of Chemistry 2021

Electronic Supplementary Information

for

Nickel-cobalt oxalate as efficient non-precious electrocatalyst for improved alkaline oxygen evolution reaction

Sourav Ghosh*[†a,b], Rajkumar Jana[†c], Sagar Ganguli[a], Harish Reddy Inta[a], Gouri Tudu[a], Heramba V. S. R. M. Koppisetti[a], Ayan Datta*[c] and Venkataramanan Mahalingam*[a]

[a]Department of Chemical Sciences, Indian Institute of Science Education and Research (IISER) Kolkata, Mohanpur, West Bengal 741246, India

[b]Technical Research Centre, S. N. Bose National Centre for Basic Sciences, Block - JD, Sector-III, Salt Lake, Kolkata -700 106, India.

[c]School of Chemical Sciences, Indian Association for the Cultivation of Science, Jadavpur, Kolkata-32, India.

*E-mail: mvenkataramanan@yahoo.com; spad@iacs.res.in; sourav.g1989@gmail.com

[†] equal contribution

1.0 Experimental Section

1.1 Materials: All chemicals were analytical grade and treated without any purification.

1.2 Catalyst Synthesis:

1.2.1 Preparation of Nickel-Cobalt Oxalate ($Ni_{2.5}Co_5C_2O_4$): 1.45 g of cobalt nitrate hexahydrate [$Co(NO_3)_3.6H_2O$] and 0.725 g nickel nitrate hexahydrate [$Ni(NO_3)_3.6H_2O$] were dissolved in 50 ml of deionised water with constant stirring for 15 min at 80°C. Then, 30 ml of oxalic acid (1.512 g) aqueous solution was added drop wise manner and again stirred for 120 min. The hot-viscous solution was quenched in ice-water and kept in room temperature (30°C) for 30 min. The precipitate was centrifuged and washed with methanol. Finally, it was dried in oven at 60°C for 12 hour. For the controlled experiment, same procedure was repeated with variation in temperature, time, amount of oxalic acid and Co/Ni ratio.

1.2.2 Preparation of Nickel Oxalate (NiC_2O_4):

For the synthesis of nickel oxalate, same procedure was repeated without [$Co(NO_3)_3.6H_2O$].

1.2.3 Preparation of Cobalt Oxalate (CoC_2O_4):

For the synthesis of cobalt oxalate, same procedure was repeated without [$Ni(NO_3)_3.6H_2O$].

1.2.4 Preparation of Nickel-Cobalt Oxide ($NiCo_2O_4$): The dry sample of nickel cobalt oxalate was collected and annealed for 350°C with calcination rate of 2°C/min and dwell time for 2 hour. All the samples are abbreviated in Table S1.

1.3 Characterization:

The phase investigation was done by X-ray diffraction (XRD) study using Rigaku-Smartlab diffractometer (Cu Kα source at 35 mA and 70 kV). Thermal features was examined by differential thermal analysis (DTA) and thermogravimetry (TG) experiment (Netzsch STA 449C, Germany). The phase analysis was further supported by FTIR (Perkin-Elmer Spectrum RX1 spectrophotometer) and RAMAN (Horiba Jobin-Yvon LabRAM HR800) study. Phase purity and composition analysis was investigated by XPS measurement using Thermo Fisher Scientific system. For microstructural characterization, FESEM study was performed by SUPRA 55-VP instrument equipped with EDAX (GEMINI column technology). TEM (JEM 2100F field emission transmission electron microscope operating at 200 kV) study was carried out for further details regarding morphology and porosity. Elemental analysis was studied by ICP-AES (ARCOS, Simultaneous ICP Spectrometer manufactured by SPECTRO Analytical Instruments GmbH, Germany). Nitrogen adsorption-desorption experiment was studied for textural engineering properties such as surface area, porosity and pore size distribution. Samples were degassed at 80°C for 4 hours in vacuum and treated for analysis by Multi-point BET (Micromeritics Gemini VII-2390t) at 77K. Electrochemical activity was measured by Biologic SP300 electrochemical workstations.

1.4 Electrochemical Techniques:

First of all, 2 mg catalyst was added in 200 μL ethanol and 40 μL Nafion mixture. Then, it was kept for sonication around 15 min. Next, 60 μL homogeneous ink was drop casted each side of carbon paper (geometric surface area was 0.25 cm^2) and dried in oven at 50°C. Loading of catalyst was maintained as 4 mg/cm^2. These electrodes were treated as working electrode and used in three electrode cell measurement. Platinum (Pt) wire and Ag/AgCl (3.5 M KCl) was employed as counter electrode and reference electrode respectively. Catalytic

performance was investigated by sweep voltammetry (LSV) and cyclic voltammetry (CV) technical mode in 1(M) KOH (pH=14). Working electrodes were initially treated for 40 precondition cycle (with a scan rate 50 mV/sec) within required potential scale. Catalytic activity was performed by LSV mode (with scan rate 5 mV/sec) using iR-compensation technique from uncompensated solution resistance (R_u). Capacitance correction was also performed for estimation of overpotential value. The measurement was demonstrated in term of reversible hydrogen electrode (RHE) scale. EIS (electrochemical impedance spectroscopy) technique was studied at 1.6 V vs. RHE for complete understanding of OER performance. pH of electrolyte (12.0, 12.5, 13.0, 13.5 and 14.0) was varied for the analysis of mechanistic pathway analysis. ECSA (electrochemical surface area) calculation was carried out from of double layer capacitance (DLC), treating 0.15 (M) KPF_6 solution in CH_3CN (acetonitrile) medium as organic electrolyte and OCP (open circuit voltage) was measured w.r.t reference electrode. CV was executed with the variation of scan rates maintaining the desired potential range (±30mV) w.r.t OCP. Next, catalytic efficiency was performed by chronocoulometry technique for the calculation of Faradaic efficiency. Volume of gas was represented as the evolved gas on the top of the burets, thereby comparison was made with theoretical value. Stability of working electrode was measured by chronoamperometry method at 1.6 V for 24 hours.

1.5 Computational Details: All the spin-polarized calculations are performed within the framework of density functional theory (DFT) using the plane-wave technique as implemented in Vienna Ab Initio Simulation Package (VASP).[1] The exchange-correlation energy was accounted within the generalized gradient approximation method (GGA) parameterized by the Perdew-Burke-Ernzerhof (PBE).[2] The on-site coulomb repulsion "U" term (DFT+U method) was used to improve the description of localized Ni and Co d-electrons in respective Nickel-Cobalt oxide and oxalates with U_{eff} = 5.30 and 3.52 eV

respectively, as recommended by the previous studies.[3-6] The ion-electron interactions were treated using the projector augmented wave potential (PAW). The DFT-D2 empirical correction method proposed by Grimme was applied for describing the effect of van der Waals interactions.[7] The kinetic energy cut off was set to be 500 eV in the plane-wave expansion for all the calculations. All the structures were fully relaxed (both lattice constant and atomic position) using the conjugated gradient method and the convergence threshold was set to be 10^{-4} eV in energy and 0.01 eV/Å in force. The Brillouin zone was sampled using a 5×5×1 Monkhorst-Pack k-point mesh for geometry optimization while a higher 7×7×1 Monkhorst-Pack grid was used to calculate electronic density of states (DOS). The OH binding/adsorption energies were calculated as[8]

$$E_{BE} = E_{OH/surface} - E_{surface} - \left(E_{H_2O} - {}^{1}\!/_{2}E_{H_2}\right)$$

Where $E_{OH/surface}$ and $E_{surface}$ are the total energies of OH adsorbed and pristine surfaces respectively while E_{H_2O} and E_{H_2} are the energies of H_2O and H_2 in gas phase. In order to calculate charge transfer between adsorbed OH and catalyst surface, we performed Bader charge-population analysis.[9-10]

In order to model $NiCo_2O_4$ (311) and $NiCo_2C_2O_4$ (202) surfaces, we considered periodic slabs with 2×1 (9.68×5.72 Å^2) and 3×2 (10.44×5.22 Å^2) rectangular surface cells with 72 and 42 atoms respectively, while NiC_2O_4 (202) and CoC_2O_4 (202) surfaces were constructed with periodic 3×2 slabs of rectangular cells (10.44×5.22 Å^2) with 42 atoms. To avoid the spurious interactions between the neighbouring slabs, we have used a vacuum layer of 20 Å in the direction perpendicular to the surfaces (along Z-direction) and the nearest distance between the two adsorbed species in the adjacent supercell is ~ 6 Å.

2.0 Figures

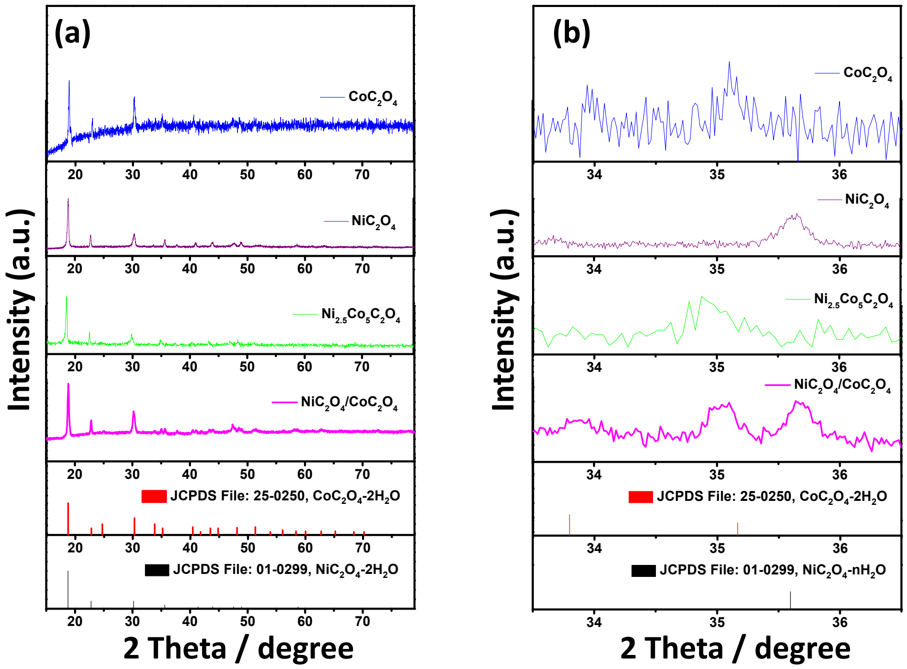

Fig. S1 (a) Low and (b) high-resolution PXRD patterns of CoC_2O_4, NiC_2O_4, $Ni_{2.5}Co_5C_2O_4$ and physical mixture (NiC_2O_4/CoC_2O_4) with standard pattern of NiC_2O_4-$2H_2O$ (ICDD No. 01-0299) and CoC_2O_4-$2H_2O$ (ICDD No. 25-0250)

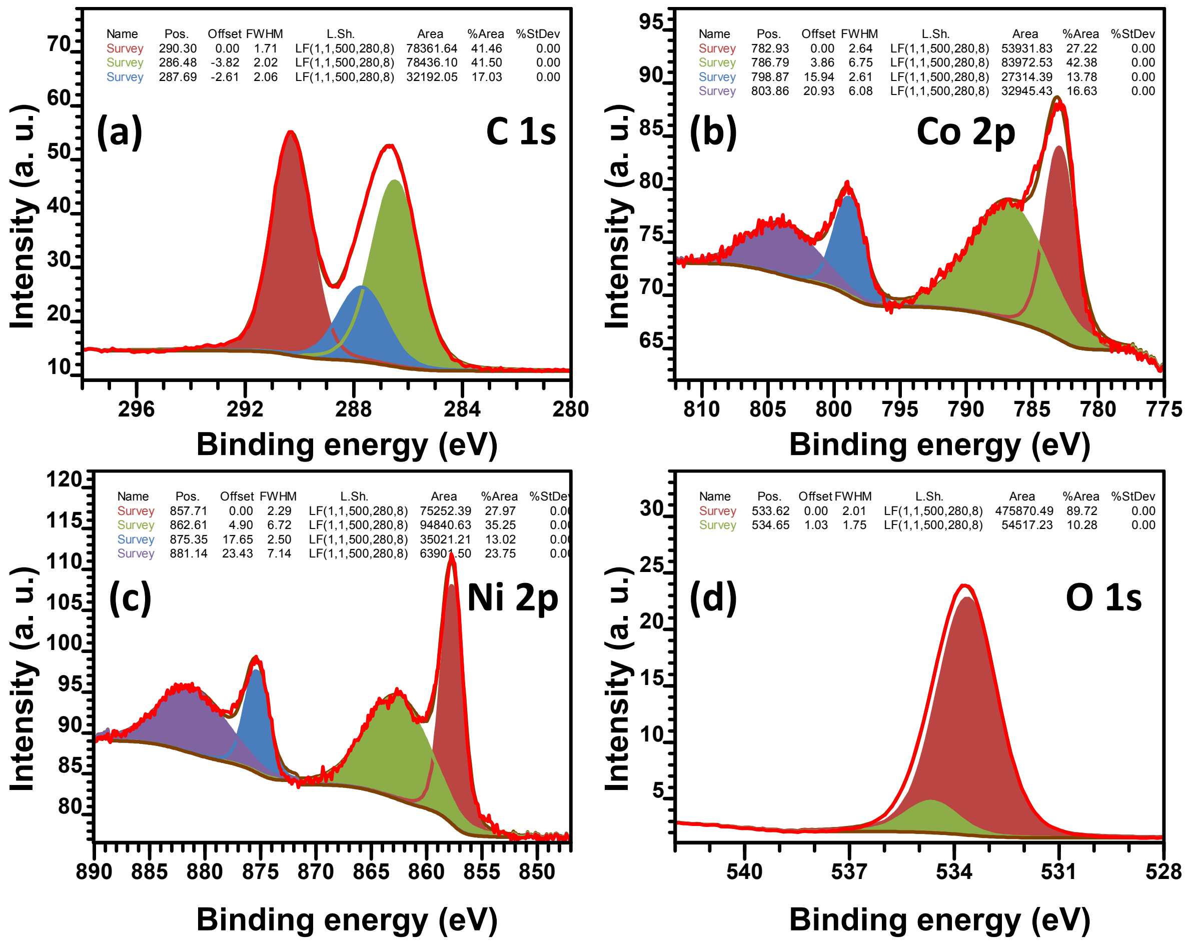

Figure S2. XPS patterns of as-prepared $Ni_{2.5}Co_5C_2O_4$ with narrow scan of (a) C1s, (b) Co 2p, (c) Ni 2p and (d) O1s.

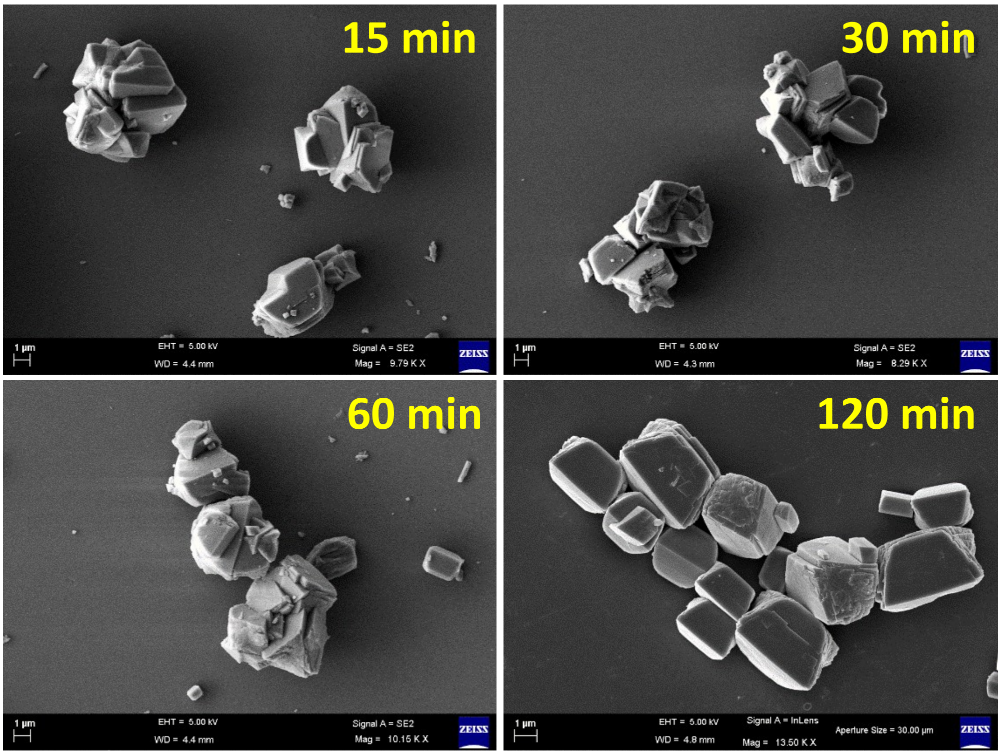

Figure S3. Time-dependent morphological evolution using FESEM study of as-prepared $Ni_{2.5}Co_5C_2O_4$ for (a) 15 min, (b) 30 min, (c) 60 min and (d) 120 min.

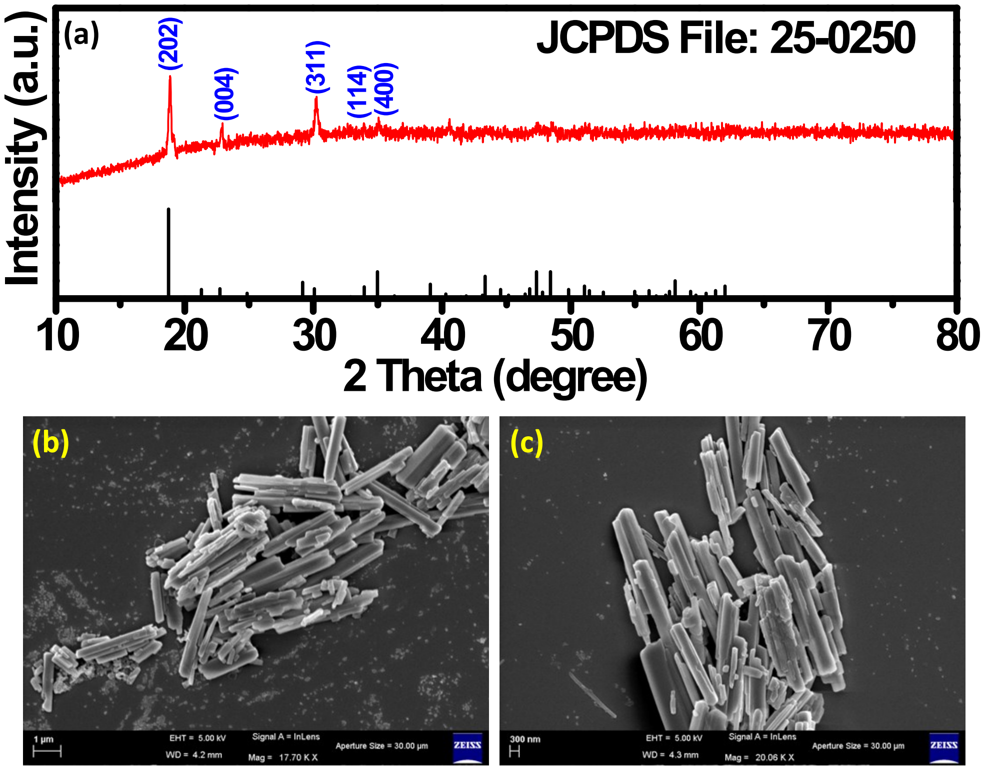

Figure S4. (a) XRD pattern and (b-c) FESEM images of as-prepared CoC_2O_4.

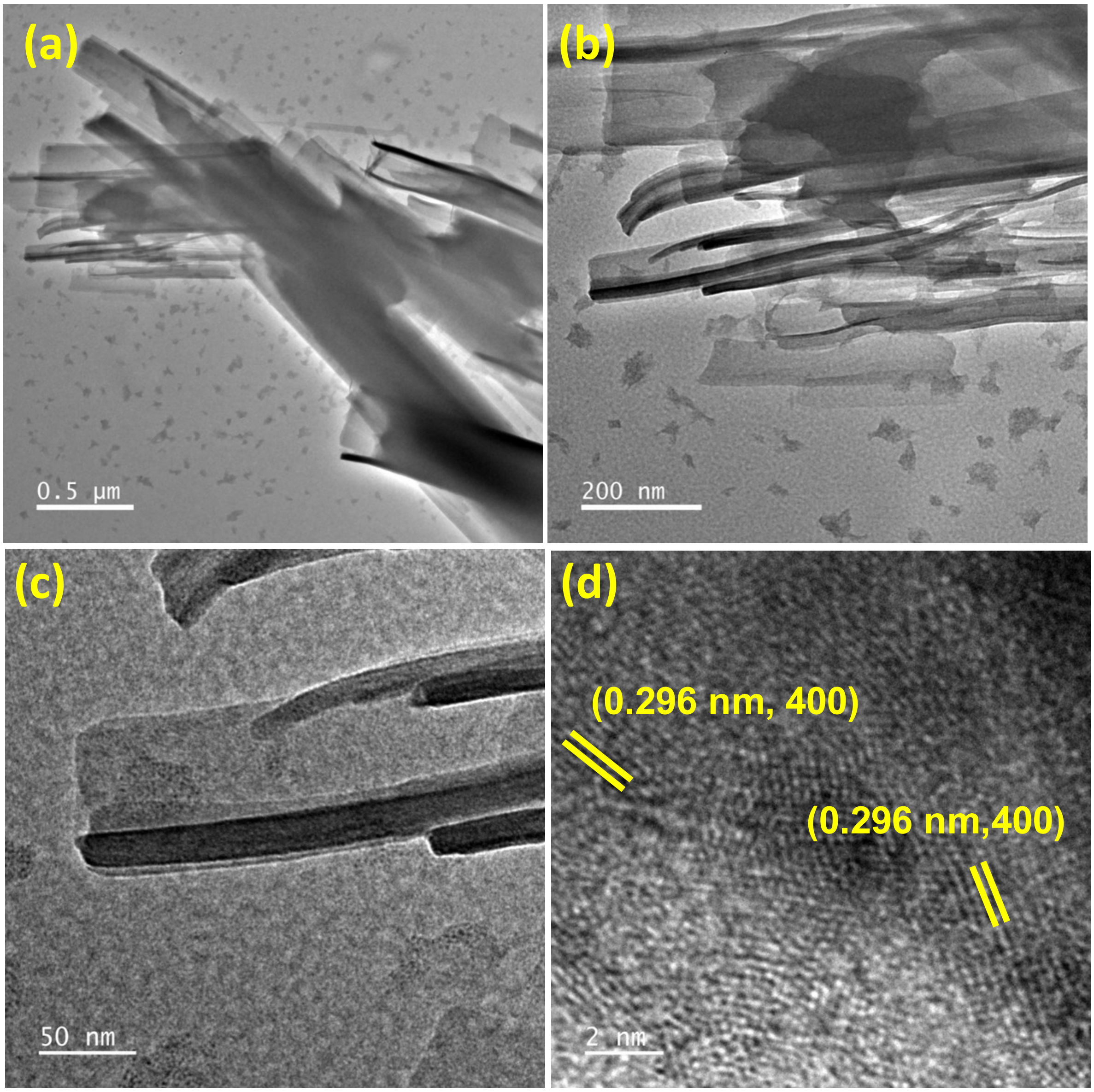

Figure S5. TEM images of as-prepared CoC_2O_4.

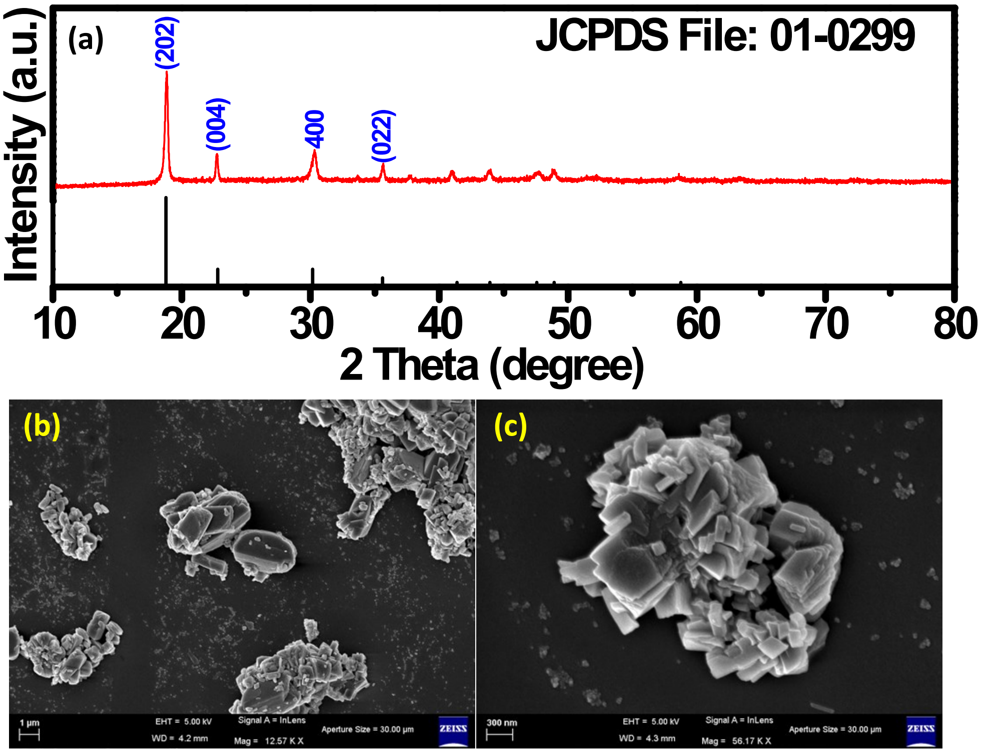

Figure S6. (a) XRD pattern and (b-c) FESEM images of as-prepared NiC_2O_4.

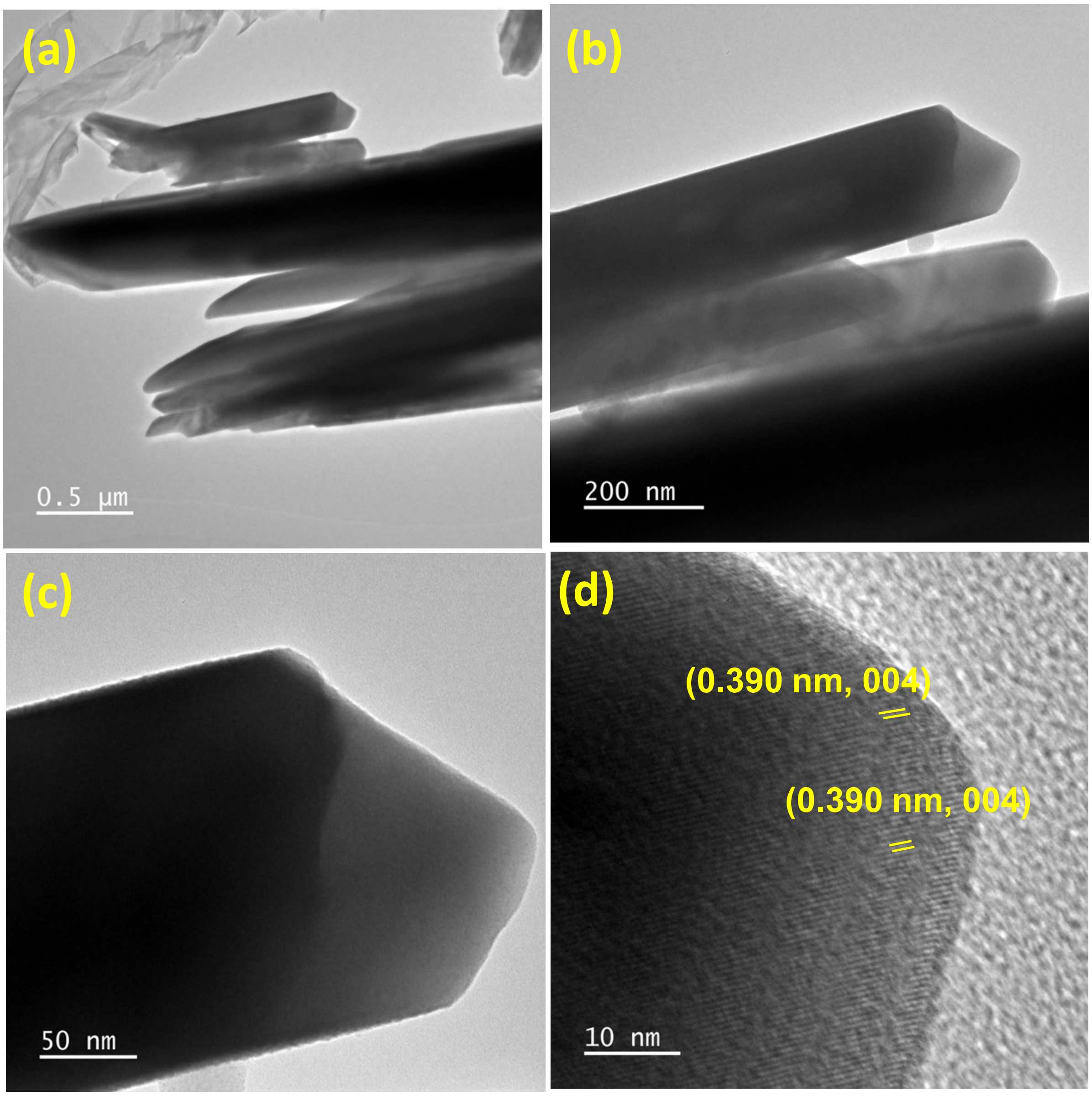

Figure S7. TEM images of as-prepared NiC_2O_4.

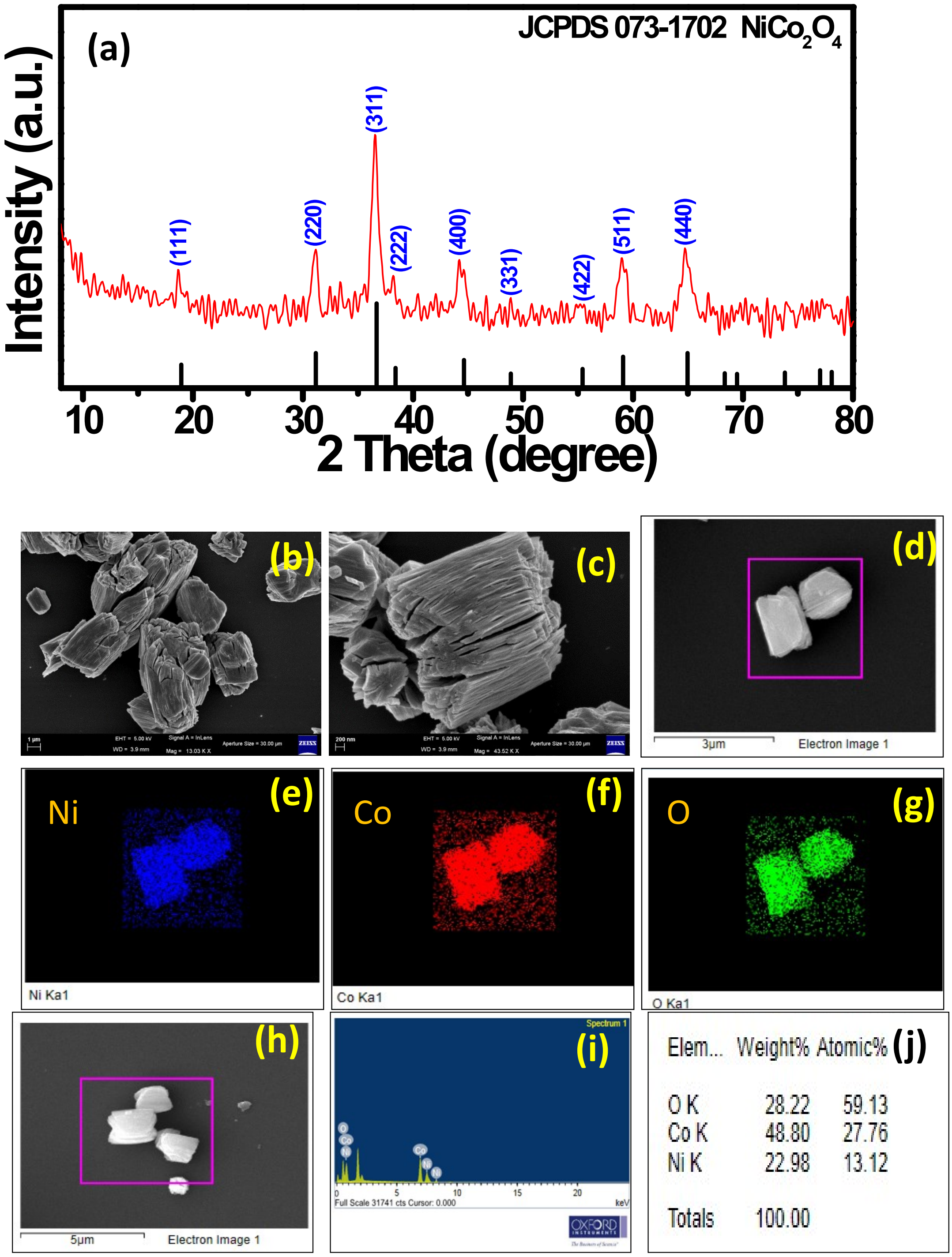

Figure S8. (a) XRD pattern, (b-c) FESEM images, (d-h) Elemental mapping and (i-j) EDS study for as-prepared $NiCo_2O_4$.

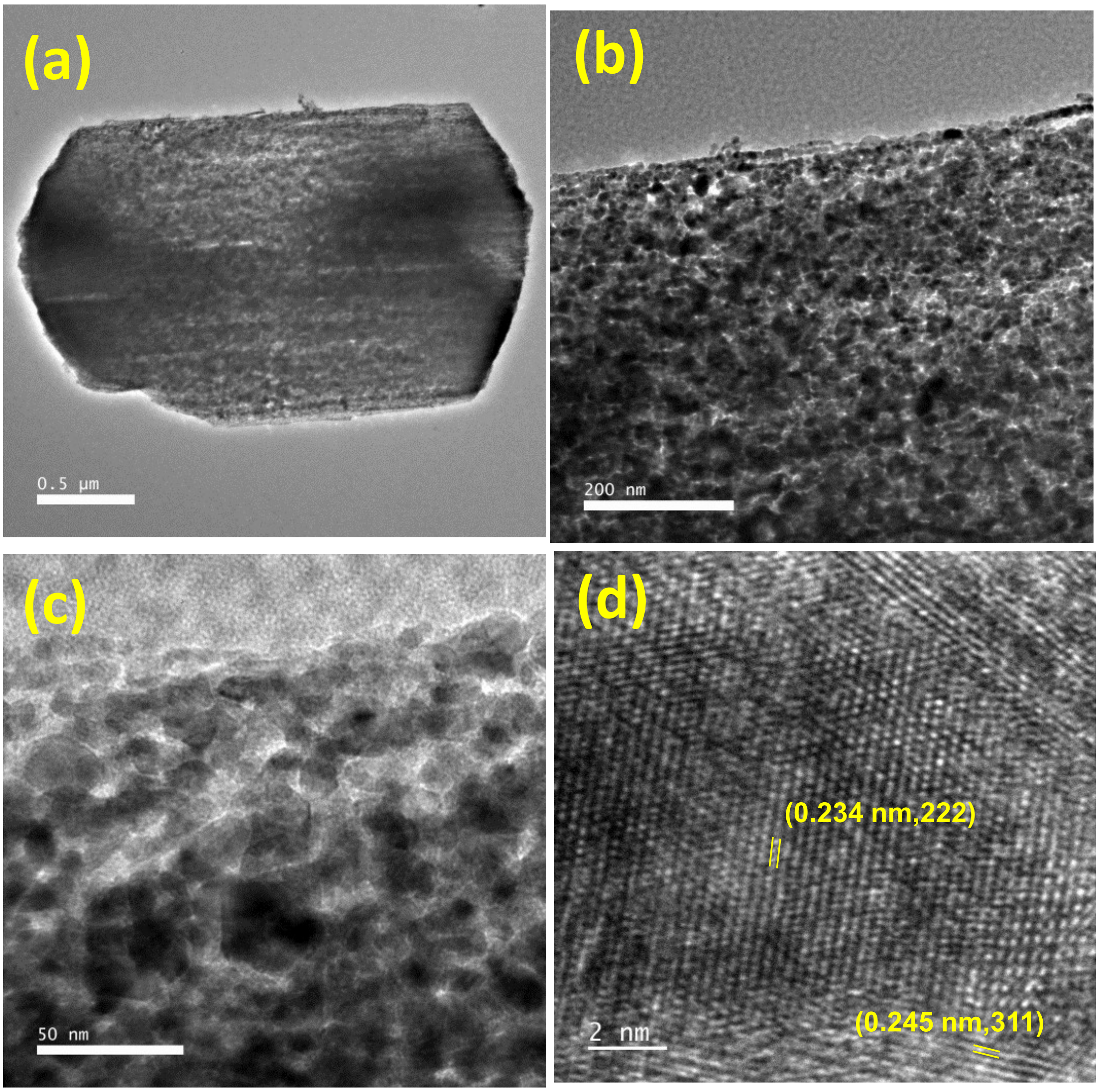

Figure S9. TEM images of as-prepared $NiCo_2O_4$.

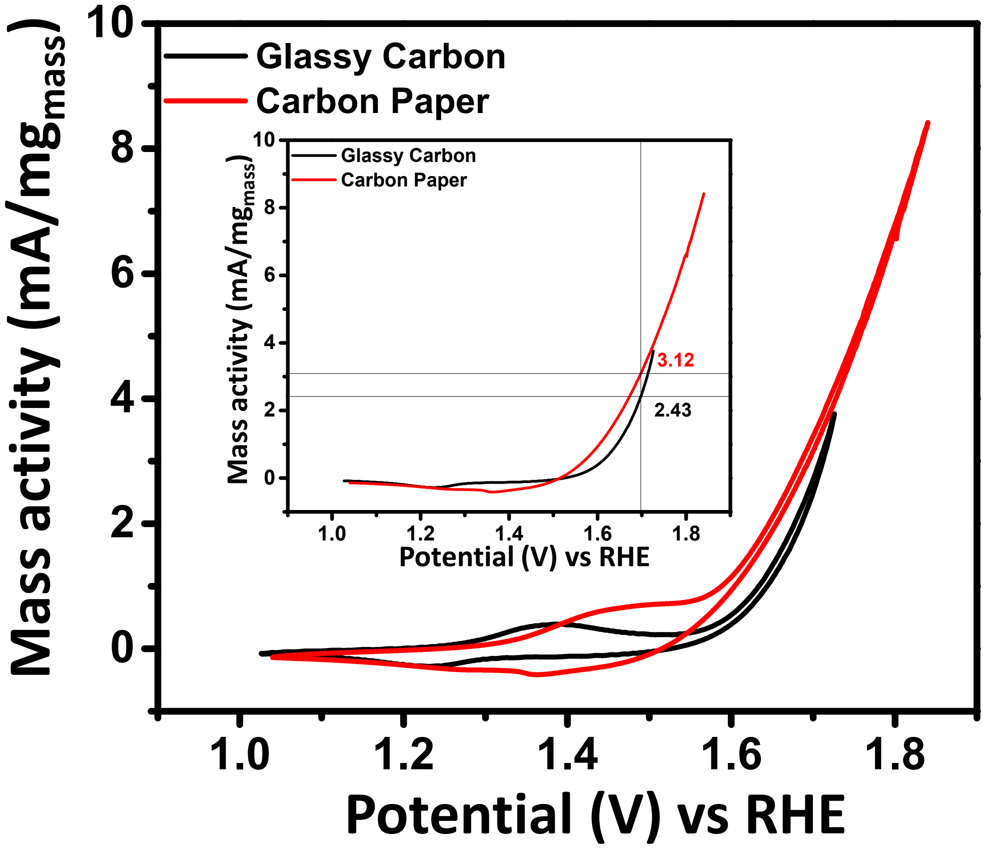

Figure S10. iR-corrected LSV scans for mass-activity of $Ni_{2.5}Co_5C_2O_4$ using carbon paper and glassy carbon as substrate for alkaline OER.

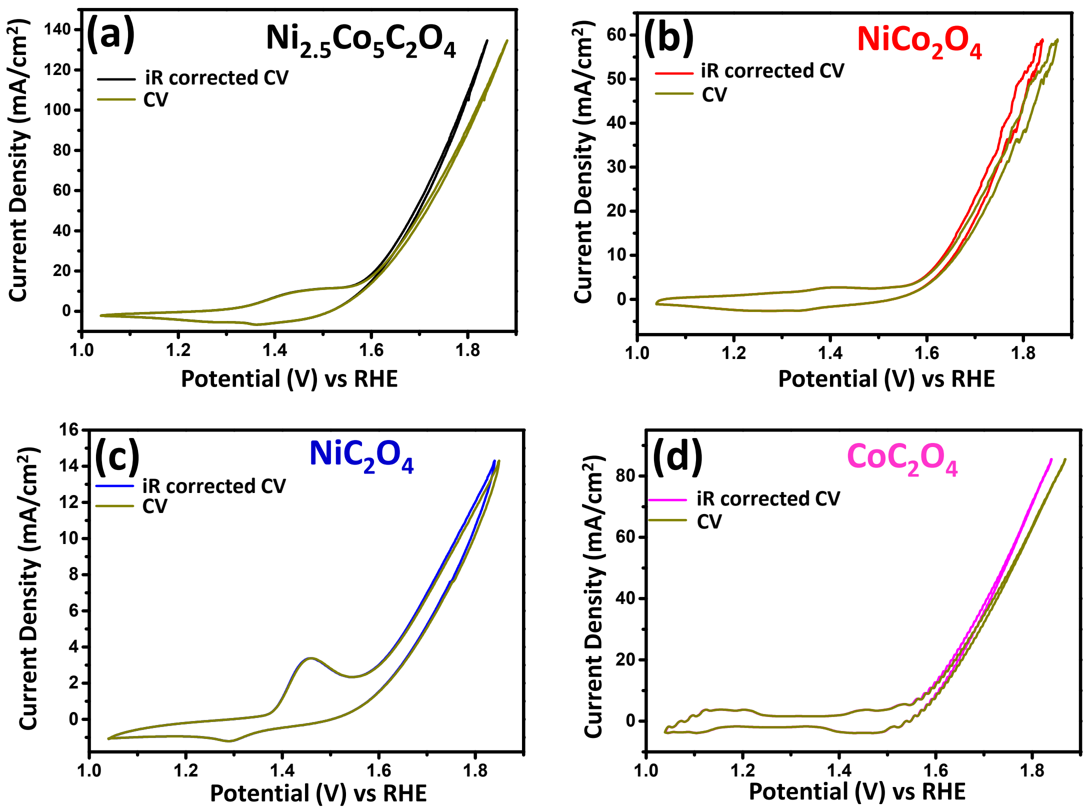

Figure S11. iR-corrected CV scans for as-prepared samples of (a) $Ni_{2.5}Co_5C_2O_4$, (b) $NiCo_2O_4$, (c) NiC_2O_4 and (d) CoC_2O_4 indicating contribution from uncompensated solution resistance (R_u).

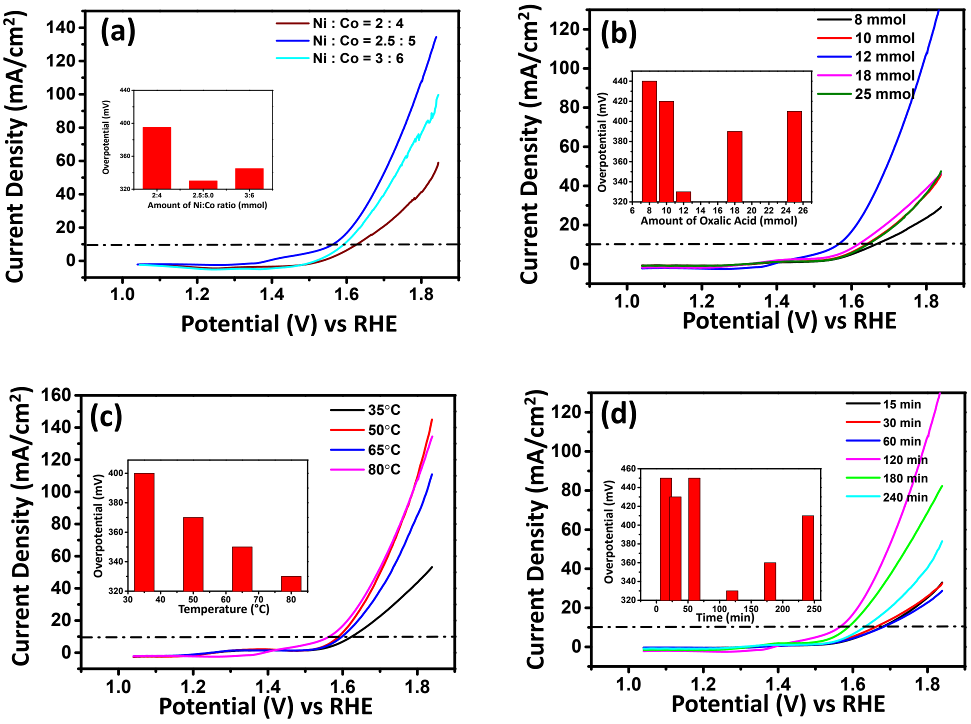

Figure S12. iR-corrected LSV scan for $Ni_{2.5}Co_5C_2O_4$ with variation of (a) Ni/Co ratio, (b) oxalic acid amount, (c) reaction temperature and (d) reaction time.

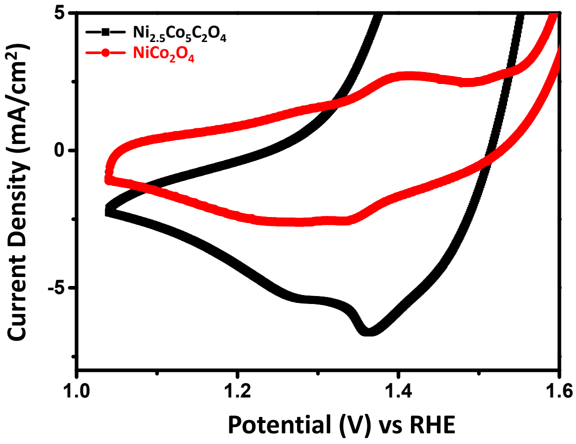

Figure S13. Higher magnification image of CV curves recorded at 5 mV/sec for as-prepared samples of $Ni_{2.5}Co_5C_2O_4$ and $NiCo_2O_4$.

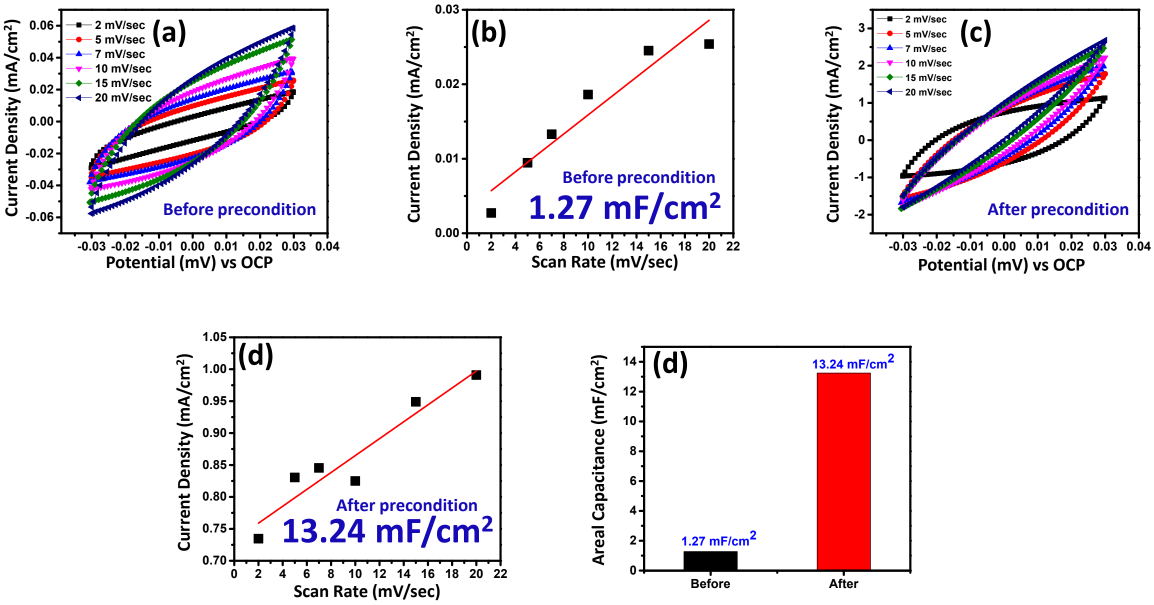

Figure S14. (a, c) CV curves recorded at different scan rates and (b, d) linear fit for estimation of double layer capacitance (C_{dl}) from DLC current vs. scan rate plots, (e) Estimation of change in double layer capacitance (C_{dl}) w.r.t before and after electrochemical precondition for CoC_2O_4 in alkaline medium (pH=14).

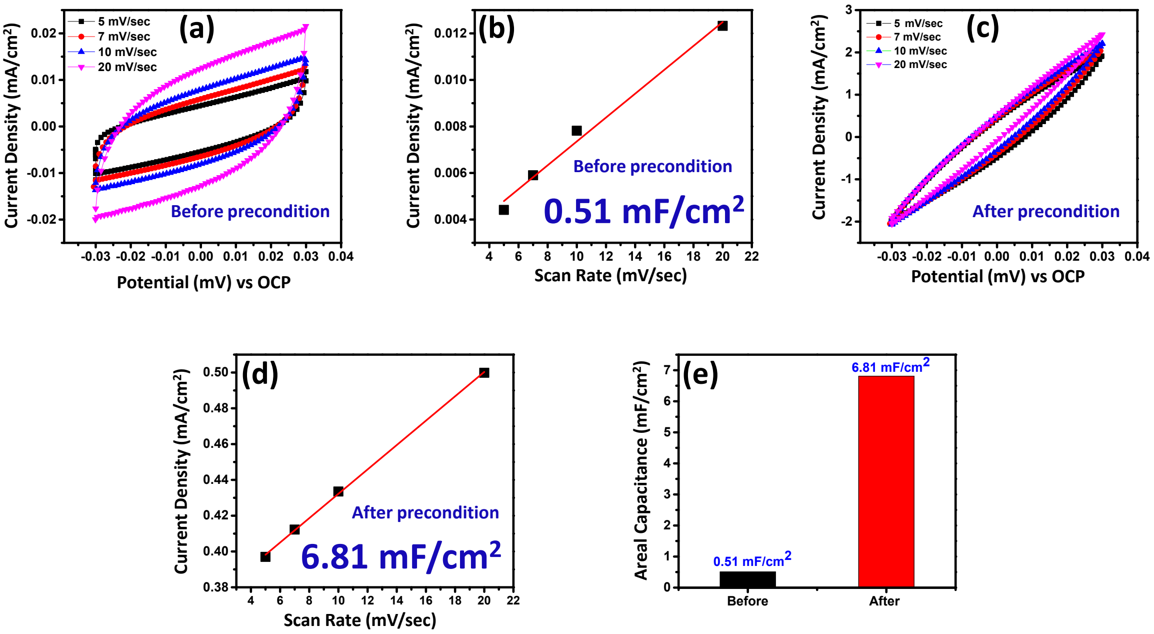

Figure S15. (a, c) CV curves recorded at different scan rates and (b, d) linear fit for estimation of double layer capacitance (C_{dl}) from DLC current vs. scan rate plots, (e) Estimation of change in double layer capacitance (C_{dl}) w.r.t before and after electrochemical precondition for NiC_2O_4 in alkaline medium (pH=14).

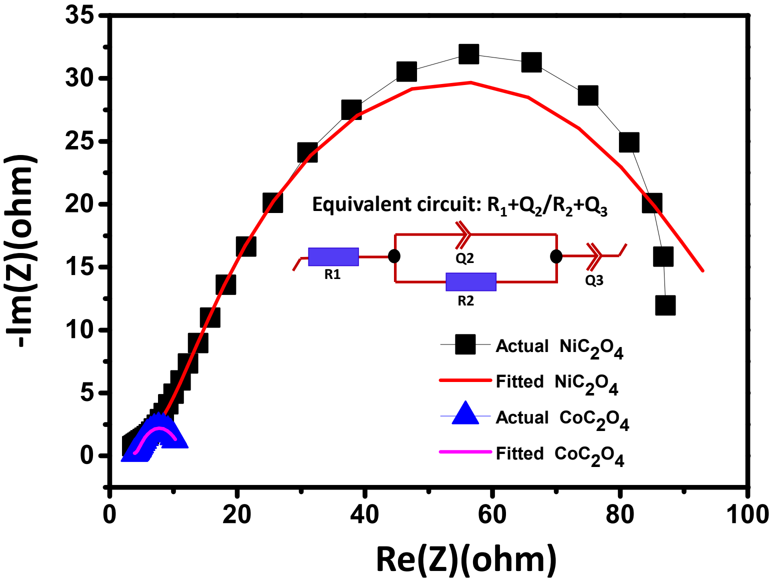

Figure S16. Nyquist plots of CoC_2O_4 and NiC_2O_4 samples.

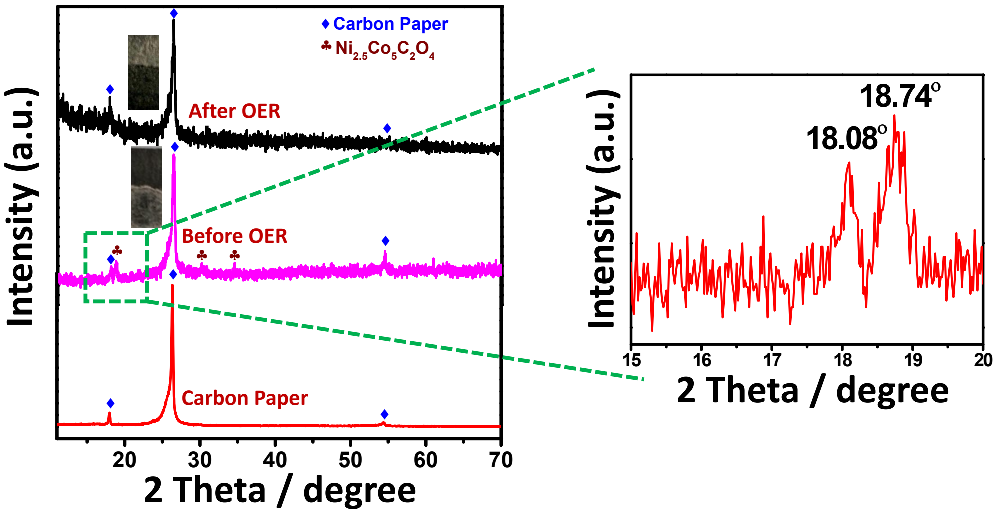

Figure S17. XRD patterns of post-catalytic $Ni_{2.5}Co_5C_2O_4$ with conspicuous peak of carbon paper.

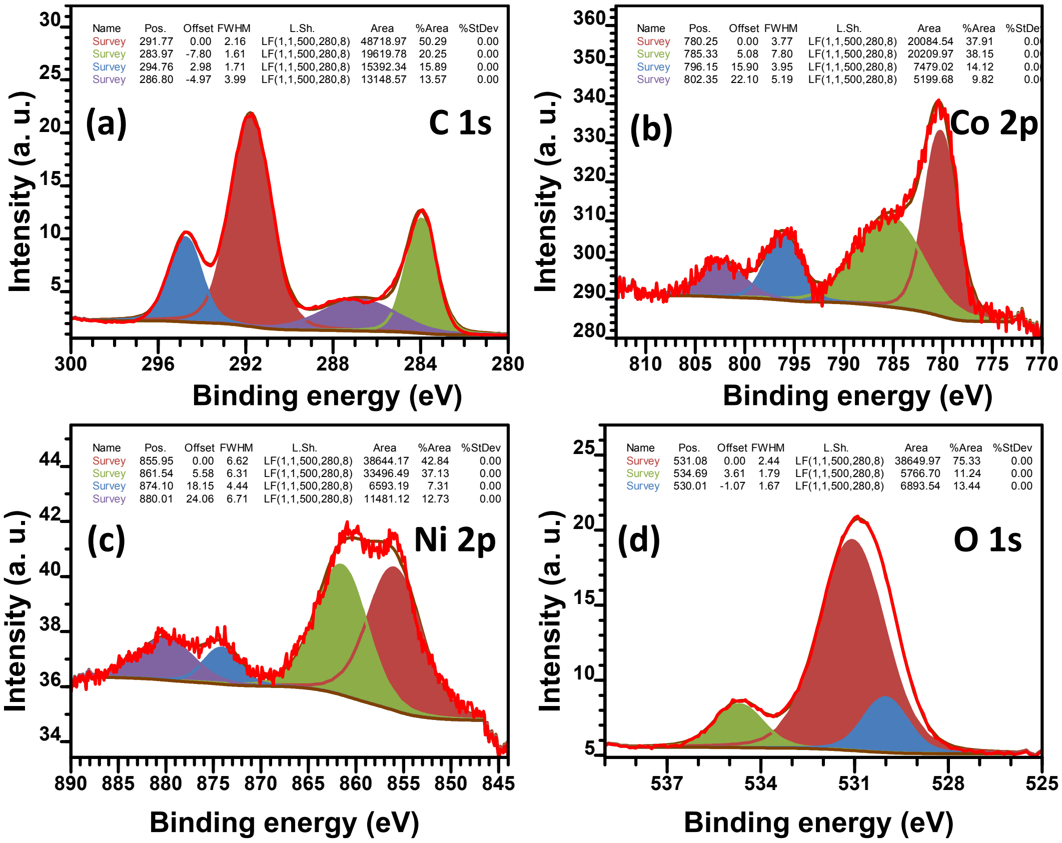

Figure S18. XPS patterns of post-catalytic $Ni_{2.5}Co_5C_2O_4$ with narrow scan of (a) C1s, (b) Co 2p, (c) Ni 2p and (d) O1s.

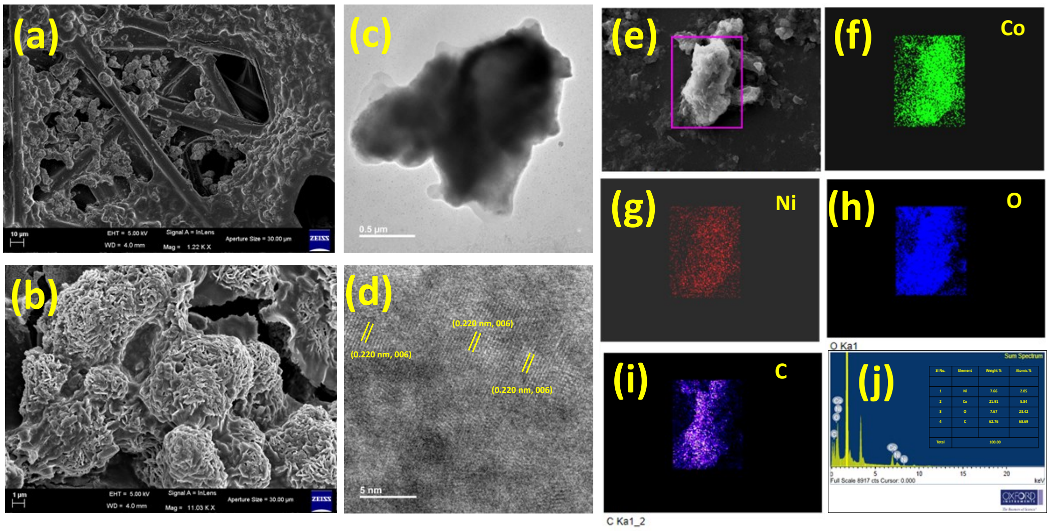

Figure S19. (a-b) FESEM, (c) TEM, (d) HRTEM images, (e-i) Elemental mapping and (j) EDS study of post-catalytic $Ni_{2.5}Co_5C_2O_4$.

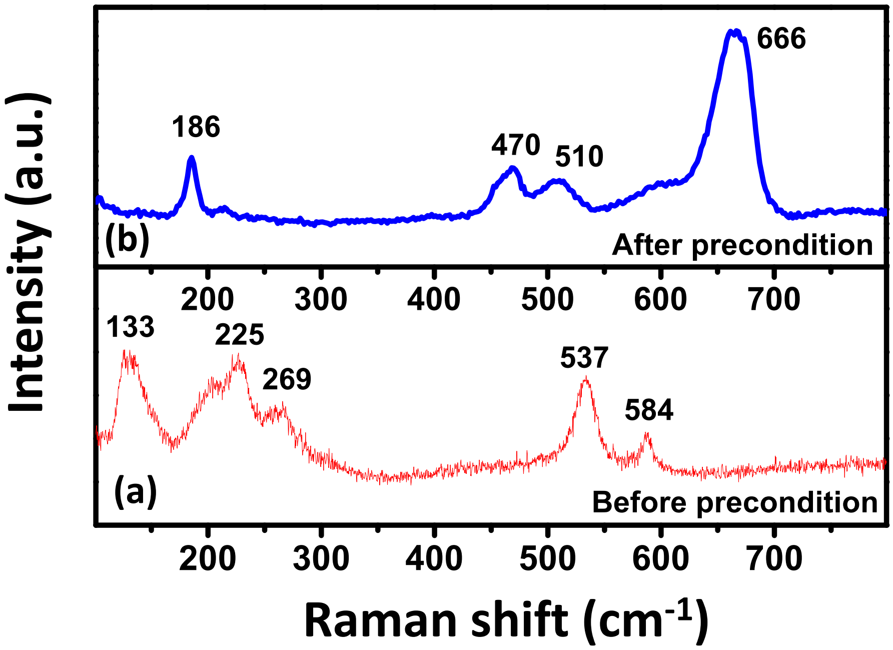

Figure S20. Raman spectra analysis of $Ni_{2.5}Co_5C_2O_4$ (a) before and (b) after precondition

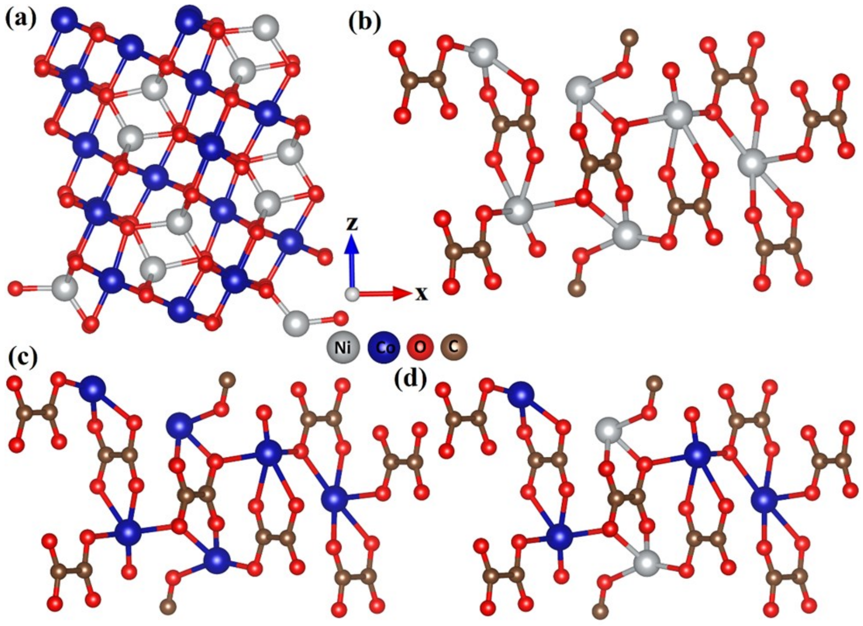

Figure S21. Optimized structure of (a) $NiCo_2O_4$ (311), (b) NiC_2O_4 (202), (c) CoC_2O_4 (202), (d) $Ni_{2.5}Co_5C_2O_4$ (202) surfaces.

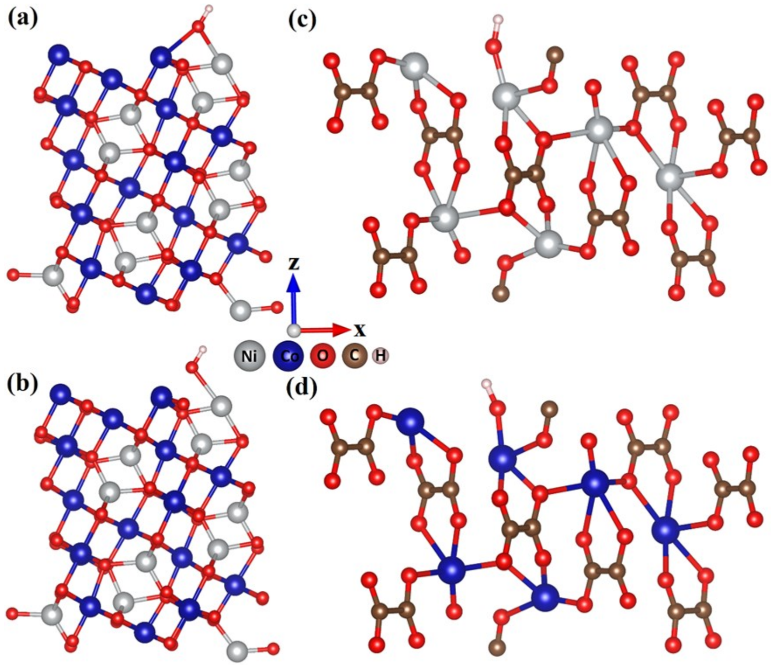

Figure S22: Optimized structures of less stable OH adsorbed $NiCo_2O_4$ (311) surfaces (a) adsorption on Ni-Co dual site, (b) adsorption on Ni-site, optimized structures of OH adsorbed (c) NiC_2O_4 (202), (d) CoC_2O_4 (202) surfaces.

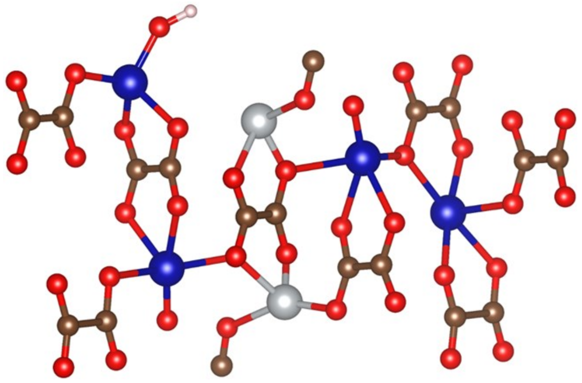

Figure S23. Optimized structures of less stable OH adsorbed $Ni_{2.5}Co_5C_2O_4$ (202) surfaces (adsorption of OH on Co^{2+}site).

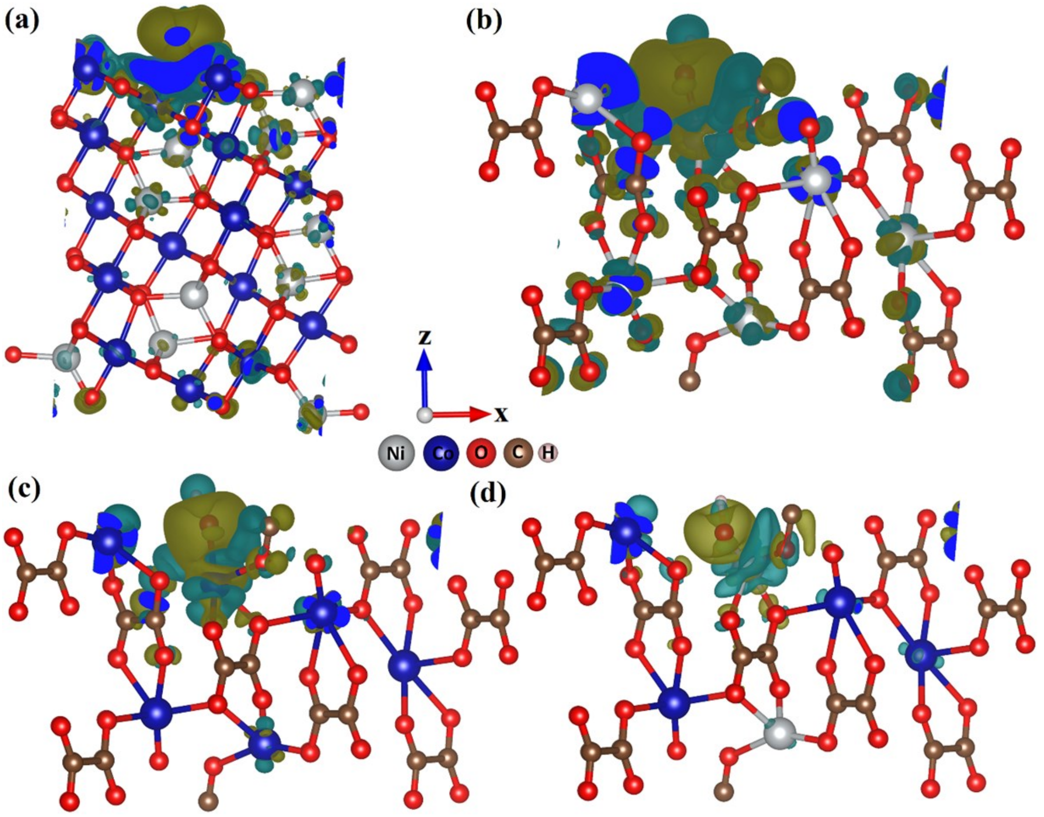

Figure S24. Charge density difference (CDD) plot (isodensity value at surfaces is ±0.002 e/au^3 (Positive: olive and Negative: cyan) of OH* adsorbed catalyst surfaces (a) $NiCo_2O_4$ (311), (b) NiC_2O_4 (202), (c) CoC_2O_4 (202), (d) $Ni_{2.5}Co_5C_2O_4$ (202).

3.0 TABLES

Table S1: The abbreviations of as-prepared samples with elemental composition.

SI No.	Sample Details	Sample ID
1	Nickel-Cobalt Oxalate	$Ni_{2.5}Co_5C_2O_4$
2	Nickel Oxalate	NiC_2O_4
3	Cobalt Oxalate	CoC_2O_4
4	Nickel Cobalt Oxide	$NiCo_2O_4$
5	Ruthenium Oxide	RuO_2

Table S2: Elemental composition of $Ni_{2.5}Co_5C_2O_4$ (working electrode) before (1-2) and after (3-4) OER process.

SI No.	Element	ppm	mg/l	Atomic Mass	mM	Ni : Co
Sample ID. $Ni_{2.5}Co_5C_2O_4$						
BEFORE OER						
1	Co	10.118	10.118	58.933	0.171	1:2.018
2	Ni	4.993	4.993	58.693	0.085	
AFTER OER						
3	Co	2.056	2.056	58.933	0.034	1:2.148
4	Ni	0.929	0.929	58.693	0.0158	
Observation: No leaching of Ni or Co was found in post-OER electrode						

Table S3: Elemental composition of $NiCo_2O_4$ (working electrode) before (1-2) and after (3-4) OER process.

SI No.	Element	ppm	mg/l	Atomic Mass	mM	Ni : Co
Sample ID. $NiCo_2O_4$						
BEFORE OER						
1	Co	21.118	21.118	58.933	0.358	1:1.977
2	Ni	10.681	10.681	58.693	0.181	
AFTER OER						
3	Co	7.733	7.733	58.933	0.131	1:1.988
4	Ni	3.931	3.931	58.693	0.066	
Observation: No leaching of Ni or Co was found in post-OER electrode						

Table S4: Study of geometric alkaline OER catalytic efficiency w.r.t Over Potential (mV) at 10 mA/cm^2_{geo} for $Ni_{2.5}Co_5C_2O_4$ with different loadings on carbon paper.

SI No.	Sample Composition	Loading density of sample (mg/cm^2)	Over Potential (mV) at 10 mA/cm^2_{geo}
	$Ni_{2.5}Co_5C_2O_4$		
1		0.664	480
2		1.332	470
3		2.664	400
4		**4.0**	**330**
5		5.332	480
Observation	Best performance was achieved for 4.0 mg/cm^2 catalyst loading		

Table S5: Study of geometric alkaline OER catalytic efficiency w.r.t Over Potential (mV) at 10 mA/cm^2_{geo} and Tafel Slope (mV/dec) for the samples.

SI No.	Sample Composition	Over Potential (mV) at 10 mA/cm^2_{geo}	Tafel Slope (mV/dec)
1	$Ni_{2.5}Co_5C_2O_4$	330	81
2	$NiCo_2O_4$	410	121
3	CoC_2O_4	370	90
4	NiC_2O_4	540	229
5	RuO_2	350	79

Table S6a: Study of geometric alkaline OER catalytic efficiency w.r.t Over Potential (mV) at 10 mA/cm^2_{geo} for different Ni/Co ratio.

SI No.	Ni (mmol)	Co (mmol)	Oxalic Acid (mmol)	Reaction Temperature (°C)	Reaction Time (mins)	Over Potential (mV) at 10 mA/cm^2_{geo}
1	0.5	1	12	80	120	490
2	1.5	3	12	80	120	440
3	**2.5**	**5**	**12**	**80**	**120**	**330**
4	4	8	12	80	120	345
5	5	10	12	80	120	380
Observation		Best performance was achieved for Ni/Co of about 2.5/5				

Table S6b: Study of geometric alkaline OER catalytic efficiency w.r.t Over Potential (mV) at 10 mA/cm^2_{geo} for different Ni/Co ratio.

SI No.	Ni (mmol)	Co (mmol)	Oxalic Acid (mmol)	Reaction Temperature (°C)	Reaction Time (mins)	Over Potential (mV) at 10 mA/cm^2_{geo}
2	2	4	12	80	120	395
3	**2.5**	**5**	**12**	**80**	**120**	**330**
4	3	6	12	80	120	345
Observation		Best performance was achieved for Ni/Co of about 2.5/5				

Table S7: Study of geometric alkaline OER catalytic efficiency w.r.t Over Potential (mV) at 10 mA/cm^2_{geo} for different Oxalic acid amount.

SI No.	Ni (mmol)	Co (mmol)	Oxalic Acid (mmol)	Reaction Temperature (°C)	Reaction Time (mins)	Over Potential (mV) at 10 mA/cm^2_{geo}
1	2.5	5	8	80	120	440
2	2.5	5	10	80	120	420
3	**2.5**	**5**	**12**	**80**	**120**	**330**
4	2.5	5	18	80	120	390
5	2.5	5	25	80	120	410
Observation		Best performance was achieved using 12 mmol oxalic acid				

Table S8: Study of geometric alkaline OER catalytic efficiency w.r.t Over Potential (mV) at 10 mA/cm^2_{geo} for different reaction temperature.

SI No.	Ni (mmol)	Co (mmol)	Oxalic Acid (mmol)	Reaction Temperature (°C)	Reaction Time (mins)	Over Potential (mV) at 10 mA/cm^2_{geo}
1	2.5	5	12	35	120	400
2	2.5	5	12	50	120	370
3	2.5	5	12	65	120	350
4	**2.5**	**5**	**12**	**80**	**120**	**330**
Observation	Best performance was achieved at 80°C					

Table S9: Study of geometric alkaline OER catalytic efficiency w.r.t Over Potential (mV) at 10 mA/cm^2_{geo} for different reaction temperature.

SI No.	Ni (mmol)	Co (mmol)	Oxalic Acid (mmol)	Reaction Temperature (°C)	Reaction Time (mins)	Over Potential (mV) at 10 mA/cm^2_{geo}
1	2.5	5	12	80	15	450
2	2.5	5	12	80	30	430
3	2.5	5	12	80	60	450
4	**2.5**	**5**	**12**	**80**	**120**	**330**
5	2.5	5	12	80	180	360
6	2.5	5	12	80	240	410
Observation	Best performance was achieved for the 120 min reaction period					

Table S10: Comparative study of geometric alkaline OER catalytic efficiency with recent literatures only for non-precious non-oxide electrocatalyst.

SI No.	Electrode material	Over potential value (mV) at 10 mA/cm^2_{geo}	Reference	Reference No.
1	$(Co_{0.7}Fe_{0.3})_2B$	330	*ChemSusChem* **2018**, *11*, 3150-3156.	12
2	Co_4N	257	*Angew. Chem.* **2015**, *127*, 14923-	9

			14927.	
3	CoC_2O_4, $2H_2O$	436 (Micro-rods) 492 (Micro-polyhedrons)	*J. Mater. Chem. A* **2015**, *3*, 9707-9713.	19
4	$(Co_xFe_{1-x})_2P$	370	*Nanoscale* **2016**, *8*, 3244-3247.	11
5	$Co_3O_4/Co_{0.85}Se/Co_9Se_8$	330	*Inorg. Chem.* **2020**, *59*, 17326-17339	10
6	Hydrous cobalt phosphate	292	*CrystEngComm.* **2019**, *21*, 884-893.	14
7	Nickel-cobalt-fluoride	300	*Chem. Commun.* **2018**, *54*, 6204-6207.	13
8	$CoWO_4$	450	*RSC Adv.* **2017**, *7*, 45615-45623.	39
9	Nickel-Cobalt Phosphide	360	*J. Mater. Chem. A* **2016**, *4*, 7549-7554.	40
10	$CoMoO_4$	343	*Chem. Phys. Lett.* **2017**, *675*, 11-14.	41
11	$Co_{0.8}V_{0.2}OOH$	190	*J. Mater. Chem. A* **2019**, *7*, 21911-21917.	42
12	Nickel-Cobalt Oxalate ($Ni_{2.5}Co_5C_2O_4$)	330	This work	-

Note: Overpotential for 10 mA/cm^2_{geo}

Table S11: Calculated OH binding energies on different catalyst surfaces.

Catalyst Surfaces	OH binding sites	OH binding energies (eV)
$NiCo_2O_4$ (311)	Co-site	-0.65
	Ni-site	-0.38
	Ni-Co dual site	-0.58
NiC_2O_4 (202)	Ni-site	-1.15
CoC_2O_4 (202)	Co-site	-0.46
$Ni_{2.5}Co_5C_2O_4$ (202)	Ni-site	-0.23
	Co-site	-0.16

4.0 References

(1) G. Kresse and J. Hafner, Ab initio molecular dynamics for liquid metals, *Phys. Rev. B.,* 1993, **47**, 558.

(2) J. P. Perdew, K. Burke and M. Ernzerhof, Generalized gradient approximation made simple, *Phys. Rev. Lett.,* 1996, **77**, 3865.

(3) R. Jana, C. Chowdhury, S. Malik and A. Datta, Pt/Co_3O_4 Surpasses Benchmark Pt/C: An Approach Toward Next Generation Hydrogen Evolution Electrocatalyst, *ACS Appl. Energy Mater.,*2019, **2**, 5613.

(4) M. García-Mota, M. Bajdich, V. Viswanathan, A. Vojvodic, A. T. Bell and J. K. Nørskov, Importance of correlation in determining electrocatalytic oxygen evolution activity on cobalt oxides, *J. Phys. Chem. C,* 2012, **116**, 21077.

(5) P. Bandyopadhyay, R. Jana, K. Bhattacharyya, O. I. Lebedev, U. Dutta, U. Sarkar, A. Datta and M. Seikh, Interaction of a bioactive molecule with surfaces of nanoscale transition metal oxides: experimental and theoretical studies, *New J. Chem.,* 2019, **43**, 16621.

(6) A. M. Ferrari, C. Pisani, F. Cinquini, L. Giordano and G. Pacchioni, Cationic and anionic vacancies on the NiO(100) surface: DFT+U and hybrid functional density functional theory calculations, *J. Chem. Phys.,* 2007, **127**, 174711.

(7) S. Grimme, Semiempirical GGA-type density functional constructed with a long-range dispersion correction, *J. Comput. Chem.,* 2006, **27**, 1787.

(8) R. Subbaraman, D. Tripkovic, K. -C. Chang, D. Strmcnik, A. P. Paulikas, P. Hirunsit, M. Chan, J. Greeley, V. Stamenkovic and N. M. Markovic, Trends in activity for the water electrolyser reactions on 3d M(Ni,Co,Fe,Mn) hydr(oxy)oxide catalysts, *Nat. Mater.,* 2012, **11**, 550.

(9) R. F. W. Bader, Atoms in Molecules: A Quantum Theory (Oxford University Press, Oxford, 1990).

(10) G. Henkelman, A. Arnaldsson and H. Jónsson, A fast and robust algorithm for Bader decomposition of charge density, *Comput. Mater. Sci.*, 2006, **36**, 354.
